# Mesothelial cell responses to acute appendicitis or small bowel obstruction reactive ascites: Insights into immunoregulation of abdominal adhesion

**DOI:** 10.1371/journal.pone.0317056

**Published:** 2025-01-08

**Authors:** Melissa A. Hausburg, Kaysie L. Banton, Christopher D. Cassidy, Robert M. Madayag, Carlos H. Palacio, Jason S. Williams, Raphael Bar-Or, Rebecca J. Ryznar, David Bar-Or

**Affiliations:** 1 Trauma Research, Swedish Medical Center, Englewood, Colorado, United States of America; 2 Trauma Research, Wesley Medical Center, Wichita, Kansas, United States of America; 3 Trauma Services, Lutheran Hospital, Wheat Ridge, Colorado, United States of America; 4 Trauma Research, South Texas Health System McAllen, McAllen, Texas, United States of America; 5 Trauma Services, Swedish Medical Center, Englewood, Colorado, United States of America; 6 Emergency Services, Wesley Medical Center, Wichita, Kansas, United States of America; 7 Trauma Services, St. Anthony Hospital, Lakewood, Colorado, United States of America; 8 Trauma Services, South Texas Health System McAllen, McAllen, Texas, United States of America; 9 Department of Molecular Biology, Rocky Vista University, Parker, Colorado, United States of America; University of Texas Medical Branch at Galveston, UNITED STATES OF AMERICA

## Abstract

Previous abdominal surgery (PAS) increases risk of small bowel obstruction (SBO) due to adhesions, and appendectomy (appy) is an independent risk factor for abdominal adhesion-related complications. Peritoneal inflammation, e.g., acute appendicitis (AA), causes formation of reactive ascitic fluid (rA) that activates peritoneum surface mesothelial cells (MCs) to form adhesions. Pathologic adhesions may arise if restoration of MC-regulated fibrinolysis and secretion of glycocalyx (GCX) are disrupted. Proteins affecting these processes may originate from peritoneal rA. This is a prospective observational IRB-approved study at three Level 1 trauma centers where rA is collected prior to surgical intervention for non-perforated AA or adhesiolysis for SBO. Samples from 48 appy and 15 SBO patients were used to treat human MCs and subjected to quantification of 85 inflammatory mediators. Results were compared between patients with surgically naïve abdomens (naïve) and patients with >1 PAS. Select rA caused MCs to form clusters of fibroblastic cells, extracellular matrix fibers (FIB), and secretion of GCX. PAS and naïve patient rA fluids were clustered into “fiber-GCX” (FIB-GCX) groups: highFIB-highGCX, highFIB-lowGCX, noFIB-highGCX, noFIB-lowGCX, and noFIB-noGCX. Between groups, 26 analytes were differentially abundant including innate immune response, wound healing, and mucosal defense proteins. Factors that contributed to the differences between groups were rA-induced highFIB and history of PAS. Overall, PAS patient rA showed a muted immune response compared to rA from naïve patients. Our data suggest that abdominal surgery may negatively impact future immune responses in the abdomen. Further, quantifying immunomodulators in peritoneal rA may lead to the development a personalized approach to post-surgical adhesion treatment and prevention.

## Background

Postoperative peritoneal adhesions are common in abdominal surgery patients [1] and can cause small bowel obstructions (SBO), infertility in women, pain, and complications in subsequent abdominal surgeries [2]. Pathologic abdominal and pelvic adhesions represent a significant economic burden, estimated at $5 billion in total inpatient expenditures per year [3].

The peritoneum that lines the body cavity, covers the viscera, and regulates peritoneal fluid, plays a significant role in adhesion regulation, and the outermost layer is an epithelial-like layer of mesothelial cells (MCs) [2, 4]. MCs produce a pericellular glycocalyx (GCX) containing glycolipids and glycoproteins that forms a smooth non-adhesive surface [5]. Surface MCs are in direct contact with peritoneal fluid and also are found free floating with resident lymphocytes and macrophages [6].

Surgical or traumatic injury to the peritoneum causes resident peritoneal macrophages to participate alongside MCs and resident fibroblasts to recruit peripheral immune cells and form adhesions [7–10]. As part of normal healing, MCs initiate adhesions by secreting fibrin and fibrinolysis inhibitors [11]. In a murine abdominal injury model, inhibition of MCs resolved peritoneal adhesions, demonstrating that MCs are central to adhesion regulation [12]. Resolution of injury and a return to peritoneal homeostasis requires fibrinolysis of adhesions, and pathologic adhesions may form following incomplete fibrinolysis and are thought to be the result of chronic inflammation and tissue remodeling [11, 13–15].

Two independent risk factors for adhesion-related rehospitalization are female sex and previous appendectomy (appy) surgery [16]. During appy, a variable amount of reactive ascitic exudate (rA) and rudimentary de novo adhesions are observed surrounding the inflamed non-perforated appendix. Surgeons report that when an appendix has perforated, extensive abdominal adhesions are often observed upon entry into the abdomen. Further, case reports describe adhesion formation and adhesive SBO in patients with parasitic infections and no previous abdominal surgeries (naïve abdomens) [17–19]. Moreover, women with endometriosis and pelvic inflammation, versus women without, are at higher risk for pathologic abdominal and pelvic adhesions [20].

These data support the hypothesis that abdominal adhesions form as a response to inflammation, independent of surgical injury. The question remains as to what part does prior abdominal inflammation versus surgically induced tissue injury play in post-surgical adhesions. Pathologic post-surgical adhesions may form in the matter of weeks or may take years to arise post-surgery [16]. SBO due to adhesions that has failed non-operative management is treated with adhesiolysis, an invasive surgical intervention to lyse constricting adhesions [21]. In more significant cases, adhesions may lead to bowel necrosis requiring resection; the most extreme case being loss of much of the small bowel that can lead to Short Bowel Syndrome, a condition characterized by malnutrition requiring intravenous supplemental nutrition [22]. In approximately half of SBO patients with surgically naïve abdomens, the source of obstruction is found to be abdominal adhesions [23]. Causes of adhesion formation in the naïve abdomen are radiology, inflammation, benign or malignant neoplasms, and endometriosis [23, 24]. Cellular mechanisms and signaling cascades leading to abdominal adhesion formation and resolution have largely been studied in animal models, and results have been difficult to translate to humans, resulting in a paucity of effective treatment options for patients [25].

Previously, we compared a limited number of appy and SBO rA samples and found that proinflammatory proteins, Interleukin-6 (IL-6) and Interferon (IFN)-γ inducible protein 10 (IP-10/CXCL10) were increased in appy rA, accompanied by increased concentrations of known inhibitors of TNF signaling [26, 27]. Cutaneous T cell-attracting chemokine (CTACK) and metabolic biproducts of reactive oxygen and nitrogen species were higher in SBO compared to appy rA. Herein, we discover phenotypic responses of MCs treated with an expanded number of samples subjected to immunomodulator quantification. Importantly, prior to surgical intervention for appy or SBO, patient fluids were collected without lavage; thus, providing a snapshot of localized proteins representing the individualized immune response of each patient. The risk of appendectomy patients developing adhesions leading to rehospitalization was established in a 10-year follow-up study showing that time to pathologic adhesion formation is highly variable [16]. However, adhesion formation following abdominal surgery is common and highly clinically relevant; thus, we compared rA immune signatures from patients categorized as PAS versus naïve. Our results may provide a foundation for developing a personalized approach to treating or preventing abdominal adhesions.

## Materials and methods

### Patient selection and clinical sample processing

This is a non-randomized, multicenter, prospective observational clinical study approved by the HCA-HealthONE (study number: 1376471) and CommonSpirit Health Research Institute (study number: 1376446) institutional review boards. Patients ≥18 years of age with non-perforated appy or SBO were being recruited with written informed consent from three Level 1 trauma centers. Initially, study participants were recruited from a single Level 1 trauma center from 05/04/2019 to 28/02/2023. At the second and third Level 1 trauma centers to be IRB approved, patient recruitment began on 20/12/2019 and ended on 30/11/2022 for this current study analysis. Exclusion criteria are as follows: patients undergoing chemotherapy or radiation therapy, receiving treatments that hinder normal immune responses, such as immunosuppressant drugs or steroids, current pregnancy or a positive result on a pregnancy test, current inflammatory bowel disease (i.e., ulcerative colitis, Crohn’s disease, malignancy), current endometriosis or autoimmune disease, other known abdominal injury at time of admission, and past-medical history of HIV/AIDS or hepatitis. One patient did not have labs drawn prior to surgery so those values were imputed in Metaboanalyst 5.0 by k-nearest neighbors based on similar samples.

All study samples were collected during operations for acute, non-perforated appendicitis by laparoscopy or treatment of small bowel obstruction by laparotomy. Before any surgical manipulation for the primary operation is performed, any rA present was aspirated into a sterile vessel. No lavage fluid was used to aid sample collection.

Post-surgery, the fluid was transferred to the clinical laboratory for STAT processing. Briefly, the sample was spun for 10 min to remove cells and debris, aliquoted, and stored at -80°C or on dry ice until further analysis. Upon arrival at the research laboratory, samples were assessed for aliquot volumes, fluid and cell pellet color, and quality of laboratory processing. All further analysis of rA occurred at the research laboratory, unless otherwise noted. At the time this study was initiated, patient samples were chosen based on previously published criteria and sufficient sample volume [26].

### Mesothelial cell culture

The normal human mesothelial cell line, LP-9 was purchased from Coriell Institute for Medical Research (AG07086), and cultured in 5% CO_2_ in 15% fetal bovine serum (Gibco, 26140–079) in 1:1 Medium 199 (Thermo Fisher, 11150059): Ham’s Nutrient Mixture F12 (Thermo Fisher, 11765054) with 10 ng/ml epidermal growth factor (EGF) (Gibco, PHG0311L), 400 ng/ml hydrocortisone (Sigma, #H0888), GlutaMAX (Thermo Fisher, 35050061), and Penicillin-Streptomycin (Pen-Strep) (Thermo Fisher, 15140122) on gelatin-coated (Sigma, G1393) tissue culture plastic.

### Mesothelial cell model and treatment with rA

LP-9 cells were grown over 4 days in 2% FBS 1:1 Medium 199:Ham’s Nutrient Mixture F12, GlutaMax, and Pen-Strep in 96- or 24-well tissue culture plates or 8-well chamber slides (Thermo Fisher Scientific, Lab-Tek II, 154534) coated with poly-L-Lysine at a density of either 0.0625 or 0.03125 x 10^6^ cells/cm^2^. Resting MCs were treated with undiluted rA, pooled human serum (ZenBio) or 2% medium for 24 to 48h.

### Fluorescent staining

#### Lectin staining

To minimize GCX disruption, cells were fixed by adding a volume of 16% paraformaldehyde to the treatment media until the final concentration was 4% paraformaldehyde. Cells were fixed for 15min at room temperature (RT) or stored at 4°C. Fixed 24h treated cells were digested with 50U/ml hyaluronidase (Sigma, CAT# H3506) in 1x PBS pH 7.4 for 10min at 37°C. Fixed 48h-treated cells were digested with 50U/ml hyaluronidase (Sigma, CAT# H3506) in 1x PBS pH 7.4 for 30min at RT. Fixed cells with and without hyaluronidase-digestion were then stained with 1:30 Alexa Fluor™ 594 Conjugate Wheat Germ Agglutinin (WGA) (Invitrogen, CAT# W11262), 1:100 Alexa Fluor™ 488 Conjugate Concanavalin A (ConA) (Invitrogen, CAT# C11252), 1:1000 Hoechst 33342 (Invitrogen, CAT# H1399) for 20min in the dark at RT. Following lectin staining, 30min hyaluronidase treated cells were stained with 1:50 Alexa Fluor™ 647 Phalloidin (Invitrogen, CAT# A22287) for 20min at RT in the dark.

### Alcian blue quantification

Cells were washed with serum-free 1:1 Medium 199:Ham’s Nutrient Mixture F12, fixed in 96-well plates with 4% paraformaldehyde overnight at 4°C, washed with 3% acetic acid solution and incubated with Alcian blue solution, pH 2.5 (Abcam, ab150662) for 15 minutes at 37°C. To remove excess Alcian blue stain, wells were washed 3 times with 3% acetic acid solution followed by 2 times with distilled water (diH_2_O) and left to dry overnight. Alcian blue staining was solubilized by 6M guanidine-hydrochloride (GuHCl, Sigma, G-4505) and relative light absorbance of the fluid was measured at 630nm with a SpectraMax M2 (Molecular Devices).

### Image acquisition and analysis

Images were acquired with a Tucsen FL-20BW camera on a Zeiss AXIO Observer Z1 Inverted Microscope, with a Lumencor Spectra Light Engine 7. Image acquisition was done with Micro-Manager [28, 29]. Image merging and processing were done in Fiji [30].

### Immunoregulator quantification

Quantification of 71 cytokines and chemokines and 14 soluble receptors were performed using bead-based multiplex assays (EVE Technologies; HD71- and HDSCR14-multi-plex assays). Analyte reference ranges were developed by Eve Diagnostics based on HD71 assay analysis of >9000 samples from healthy and diseased EDTA-treated plasma or serum samples. Missing values were imputed in Metaboanalyst 5.0 by k-nearest neighbors. Across all samples, granulocyte-macrophage colony-stimulating factor (GM-CSF), interleukin-2 (IL-2), IL-3, IL-4, IL-7, and small inducible cytokine A1 (I-309) had median concentrations of less than 1 pg/ml and max values of less than 10 pg/ml and were removed from further analysis.

### Statistical analyses

In addition to lab-based analyses of the ascites fluid collected, a number of clinical variables were also collected on all patients enrolled in the study: demographics (age, sex), comorbidities, pre-admission medications, prior abdominal surgeries, time from symptom onset to procedure, treatments administered in the hospital (medications and fluids), total hospital length of stay, lab (e.g., white blood cell count, lymphocyte count, etc.) and chemistry (e.g., sodium, potassium, glucose, etc.) values. Chi-square, Fisher’s exact tests, Wilcoxon rank sum tests, and Kruskal Wallis with post hoc Dunn’s comparisons were used to detect differences in clinical variables and immunomodulator concentrations. Alpha <0.05 was considered statistically significant. These statistical analyses were performed in either Statistical Analysis Software (SAS, SAS Institute, Inc.) or RStudio with the programming language R [31, 32]. R packages used for analyses and graphs included: Tidyverse, readxl, Purrr, rstatix, ggpubr, and ggVennDiagram [33–38]. Hierarchical clustering and associated heat maps of immunomodulator were performed in Metaboanalyst 5.0 [39].

### Ingenuity Pathway Analysis

Data were analyzed with Ingenuity Pathway Analysis (IPA; QIAGEN Inc.). Log_2_ fold-changes were calculated by dividing median immunomodulator values for each PAS/naïve-FIB-GCX group by median values from PAS-highFIB-highGCX. IPA generated a “signaling pathway” analysis of immunomodulators that were significantly more than 2-fold differentially abundant between comparison groups (Dunn’s pwc; FDR<0.05). Graphical summaries and comparison analyses were used to visualize pathways common to each group pairwise comparison.

## Results

### Treatment of mesothelial cells with appy and SBO rA

#### Clinical characteristics of 63 patient rA-fluids tested on MCs in vitro

Treatment of MCs initially included a cohort of 48 appy and 15 SBO patient rA samples. There were no significant differences in sex within this cohort (Table 1, 46% female appy versus 67% female SBO patients, P = 0.266); however, appy patients were significantly younger than SBO patients (34 years of age versus 77, P<0.001). SBO patients undergoing adhesiolysis had higher occurrences of prior abdominal surgery (P<0.001) and had a significantly longer time between symptom onset and surgical intervention (median 87.5h versus 23.2, P = 0.006). Of the appy and SBO rA fluids, 5 samples were toxic and resulted in MC cell death within 24h of stimulation; thus, these samples were excluded from further analysis (4% appy rA were toxic versus 20% SBO rA, P = 0.1518).

**Table 1:** Cohort of 63 appy and SBO patient fluids tested on MCs in vitro.

	APPY	SBO	P
	n = 48 (76%)	n = 15 (24%)	
Sex (% female)	46% (26)	67% (5)	0.266
Age (median (IQR))	34 (26.2)	77 (23.5)	<0.001
Number of prior abdominal surgeries			<0.001
0	85% (41)	7% (1)	
1	10% (5)	27% (4)	
2	2% (1)	40% (6)	
3+	2% (1)	27% (4)	
BMI (median, IQR)	26.6 (9.1)	26 (5.6)	0.599
Symptom onset to procedure (hours, median (IQR))	23.2 (24.4)	87.5 (101.6)	0.006
Percent (number) of rA fluids that killed treated MCs after 48h in culture	4% (2)	20% (3)	0.1518

rA fluid from 48 appy and 15 SBO patients were compared for select clinical characteristics and toxicity to MCs in culture; P<0.05 was considered significant.

#### Development of an in vitro mesothelial cell model

In direct contact with peritoneal rA fluid, MCs rapidly respond to danger- and pathogen-associated molecular patterns (D/PAMPS) and inflammation. To study mesothelial cellular responses to appy or SBO rA, we developed an in vitro model where MCs were plated in 2% FBS medium (2% Control) without the addition of EGF, a powerful MC mitogen used during expansion in culture. Four days following plating, MCs grown in 2% medium supplemented with EGF showed an increased number of cells with a fibroblastic morphology compared to MCs grown in 2% Control without EGF that showed cuboidal-like morphology and less proliferation (S1A Fig).

#### Reactive ascites initiates mesothelial cells to produce extracellular matrix, mucopolysaccharides, and glycoproteins

**Fig 1:**
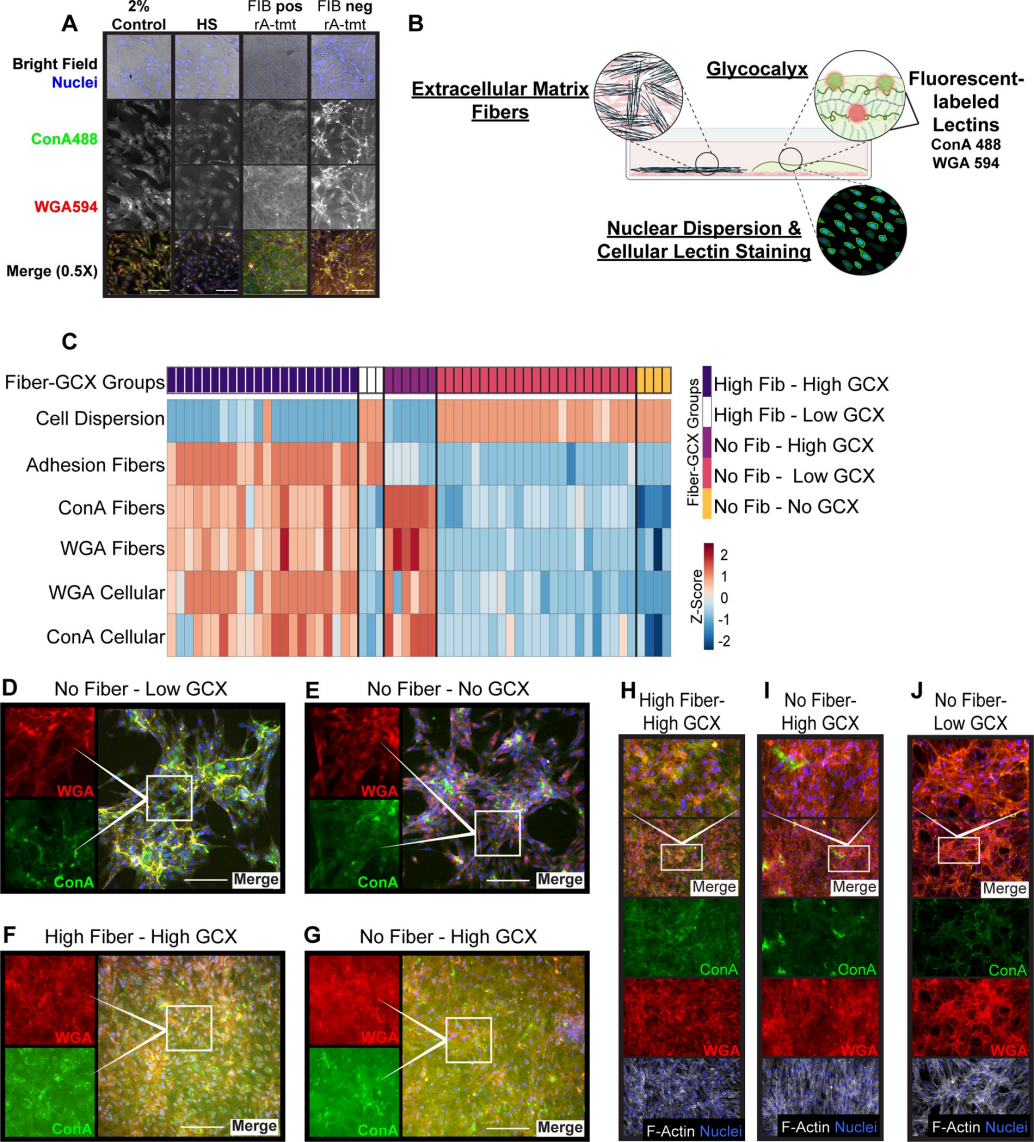
Patient rA treatment of MCs initiates formation of distinct phenotypes based on production of ECM fibers and glycocalyx secretion. (A and D–J) Fluorescent images of cells stained with wheat germ agglutinin-594 (WGA 594): red, concanavalin A-488 (ConA 488): green, Hoechst (nuclei): blue, and Phalloidin-647: white (H–J only). (A) Brightfield and fluorescent images of MCs after 24h of culturing in 2% Control medium, human serum (HS), or with rA fluid from two patients. The rA fluid on the left initiates the MCs to produce ECM fibers while the other does not. To aid in visualization of the ECM fibers, MCs in this panel were fixed and digested for 10min with hyaluronidase prior to staining. (B) Overview of the parameters that were evaluated and scored to categorize the patient rA-fluids based on the observed phenotypes displayed by treated MCs. ECM fibers were scored by brightfield microscopy. Cellular dispersion of Hoechst-stained nuclei and lectin GCX staining were scored by fluorescence microscopy (C). Heatmap of phenotype scores showing five fiber-GCX categories that 58 patient rA fluids were assigned where each column corresponds to one patient sample. Above the heatmap are boxes colored to illustrate the groups that the fluids were assigned: dark purple–high fiber-high GCX, white–high fiber-low GCX, purple–No fiber-high GCX, pink–no fiber-low GCX, and yellow–no fiber-no GCX. Each row of the heatmap shows z-scored results from at least three independent experiments where scores were averaged or added together for ECM fiber scoring. (D–G) Panels with representative images of fiber (FIB)-GCX categories: (D) noFIB-lowGCX, (E) noFIB-noGCX, (F) highFIB-highGCX, and (G) noFIB-highGCX. (H–J) Panels with representative images of FIB-GCX categories after a 30min hyaluronidase digestion that allowed for staining of f-actin: (H) highFIB-highGCX, (I) noFIB-highGCX, and (J) noFIB-lowGCX. White inset boxes indicate zoomed regions within each panel of images, and white scale bars show 100μm.

To recapitulate the intraabdominal environment of each patient as closely as possible, we replaced the culture medium of 4-day resting MC cultures with neat rA from appy or SBO samples. In a pilot analysis of 4 appy and 3 SBO patients, we observed dramatic changes in cell-cell interactions and morphology in rA-treated cells versus control conditions (S1B and S1C Fig). Morphology was assessed by brightfield, and we observed that MCs treated with select patient rA produced a layer of dense thread-like ECM fibers, insensitive to a 10 min hyaluronidase digestion (Fig 1A and 1B). We considered that ECM fiber formation and changes in morphology may be a general reaction to switching from lower protein conditions (2% Control) to high protein (neat rA fluid); thus, we treated MCs with neat human serum (HS). Culture in HS or 2% Control did not result in significant changes in cell morphology and did not induce cells to produce ECM fibers (Fig 1A).

Conceivably a separate phenomenon from the ECM fibers, we observed that mesothelial cells treated with select rA produced a transparent gelatinous GCX that we quantified using a modified-Alcian blue assay (Fig 1B and S1C Fig). Plant lectins bind to sugar-moieties on glycoproteins and other sugar-modified macromolecules. Lectins have been used to characterize the GCX and clinically, in differential cytological diagnosis of pleural effusions [40]. Normal and reactive MCs stain with multiple lectins [40, 41], and changes in lectin-binding patterns and cellular localization show that MC GCX is dynamically regulated [42]. We stained the GCX made by rA-treated MCs with the Alexa Fluor-conjugated lectins, Concanavalin A (ConA488) and wheat germ agglutinin (WGA594). ConA binds to α-D-mannosyl and α-D-glucosyl residues [43]; whereas, WGA binds to N-acetyl-D-glucosamine and Sialic acid [44]. MCs treated for 24h with either 2% control, HS, or patient rA showed positive staining for ConA and WGA, albeit at differing intensities and patterns of cellular staining (Fig 1A and 1B).

#### Characterization of ECM fibers and GCX staining in rA-treated MCs revealed four major phenotypes

Despite dissimilarities in etiologies, similar MC morphological transformations were observed after treatment with rA collected from appy and SBO patients. Patient clinical characteristics and/or signaling pathways may be shared by rA triggering each distinct MC morphology. MC morphologies were characterized in six different categories with scoring of: ECM fiber formation, cellular dispersion, abundance/characteristics of lectin-stained GCX, and the location of lectin staining within the cells (Figs S2A–S2E and 1B and 1C and S2 Table). ECM fibers were scored based on appearance and abundance at three levels: no–, low–, and high–fibers (Figs S2A and 1C, second row). Cell dispersion was scored at three levels, “evenly” distributed, “clustered” or a mix of the two (Figs S2B and 1C, first row).

Lectin-stained GCX were scored over 5 levels (S2C Fig). A score of one represented little to no extracellular staining, which was primarily observed in control treatment conditions. Level 2 represented staining where the extracellular matrix appeared broken up and granular. Levels 3 to 5 showed increasing abundance of extracellular lectin staining with a score of 5 showing diffuse staining within a gelatinous-like GCX (S2C and S2D Fig). Cellular patterns of lectin-staining were scored at three levels, level 1–no nuclear staining, 2 –staining distributed over the cell, and 3 –diffuse staining where individual cells were difficult to visualize (S2E Fig).

Patient rA-fluids were assigned to one of 5 “fiber-GCX” groups based on scoring similarities over the six categories (Fig 1C). rA-treated MCs that organized into clusters (higher Cell Dispersion score) also showed less overall GCX and no ECM fibers except for 3 patient rA-fluids that formed high fibers and low GCX (highFIB-lowGCX; Fig 1C, white bars, second group from left). rA-fluids from 23 patients elicited MCs to produce no fibers and low GCX (noFIB-lowGCX; Fig 1C, magenta bars, forth group from left; Fig 1C). Four patient rA-fluids elicited MCs to produce no fibers and no GCX (noFIB-noGCX; Fig 1C, yellow bars, fifth group from left; Fig 1E). ECM fibers were most often observed with high lectin staining and evenly distributed nuclei across the field of view (lower Cell Dispersion score; 22 patient rA-fluids; highFIB–highGCX; Fig 1C, dark purple bars, first group from left; Fig 1F); however, treatment with six of the rA samples stimulated formation of high GCX without inducing ECM fibers (noFIB–highGCX; Fig 1C, purple bars, third group from left; Fig 1G). In samples with high GCX production, the visualization of individual cell morphology was nearly impossible compared to low GCX conditions (compare Fig 1D and 1E with Fig 1F and 1G).

In samples with high GCX, attempts to interrogate cell morphology, through cytoskeletal filamentous actin (F-actin) staining with phalloidin were unsuccessful. To moderately disrupt the impenetrable GCX layer, we digested fixed samples for 30 min with hyaluronidase, which allowed access underneath the GCX layer resulting in intracellular F-actin staining in highFIB-highGCX and noFIB-highGCX samples (Fig 1H and 1I). Consistent with our observations of cellular dispersion, F-actin staining showed that in conditions associated with high GCX, MCs were generally evenly distributed and not forming extensive cluster networks as observed in noFIB-lowGCX conditions (Fig 1J). Moreover, we observed cells that may be undergoing the process of tubularization and lifting in high GCX conditions (F-actin; Fig 1H and 1I). This phenomenon is thought to be observed because of an increase in cell surface proteoglycan density causing electrostatic interactions within the GCX that deform the cell membrane [19].

### Analysis of rA fluid immunomodulating proteins with MC phenotypes and patient clinical characteristics

**Fig 2:**
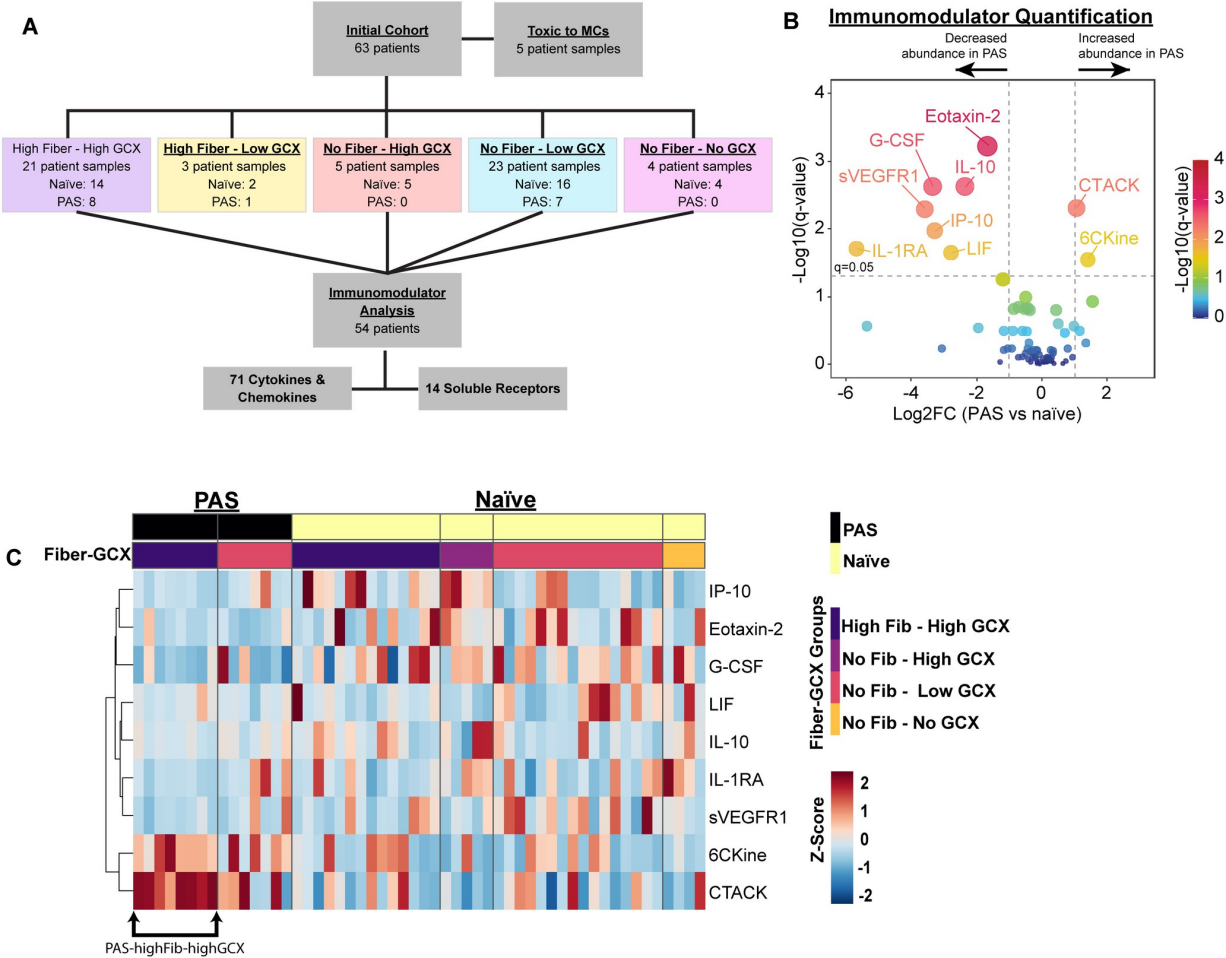
PAS patient rA shows differential abundance in 9 immunomodulator proteins when compared to rA fluid collected from naïve patients. (A) Flow-chart depicting decisions points leading to inclusion of 54 patient rA samples in the immunomodulator analysis down from the original 63-patient cohort. Five rA samples from the 63-patient cohort were toxic to MCs following 48-h of treatment and were excluded from further analysis. Thus, 57-patient rA samples were characterized, as illustrated in Figs 1 and S2, into 5 distinct FIB-GCX groups based on scoring of rA-treated MCs. Of the FIB-GCX groups, the highFIB-lowGCX group did not contain over 3 rA samples in our primary variable of interest, patient history of PAS; thus, this group was excluded from further analysis. Immunomodulator analysis of the 54-patient cohort consisted of quantification of 71 cytokines and chemokines and 14 soluble receptors. (B) Volcano plot with immunomodulators that were fold-regulated >|2|-fold (vertical dashed lines) graphed with -log_10_ transformed FDR-adjusted q-values<0.05 (horizontal dashed line) in PAS versus naïve patient rA. (C) Heatmap showing the z-scored concentrations of the proteins depicted in B in each individual patient rA displayed as columns. The rectangles above the heatmap show which group each individual sample is assigned. PAS (black) and naïve (yellow) patient samples are separated by a dashed line. The second row of rectangles shows the assigned FIB-GCX group based on cellular scoring depicted in Fig 1. PAS rA elicited either highFIB-highGCX (dark purple) or noFIB-lowGCX (magenta); whereas the naïve samples were assigned to 4 different groups: highFIB-highGCX (dark purple), noFIB-highGCX (medium purple), noFIB-lowGCX (magenta), or noFIB-noGCX (orange). The black arrowed-bracket delineates the group of PAS-highFIB-highGCX patient samples that showed a high level of congruence in immunomodulator concentration between the samples.

Treatment of MCs with rA caused changes in morphology, cellular behavior, and extracellular matrix production that correlated with distinct phenotypes. We hypothesized that distinct cellular phenotypes may be regulated by immunomodulating proteins such as cytokines, chemokines, and immunoregulatory soluble receptors. Further, immune signaling within the peritoneal cavity may be altered post-abdominal surgery resulting in disparate immune responses between patients with or without a history of previous abdominal surgery (PAS). To explore these hypotheses, we compared patient samples dependent upon history of previous abdominal surgery within each FIB-GCX group (Fig 2A). We excluded highFIB-lowGCX samples because neither PAS group had more than 3 samples; thus, four FIB-GCX groups consisting of 54 patient samples were selected for further analyses (S1 Table).

#### Clinical characteristics of selected patient rA-fluids

Patients with a history of PAS were older, more likely to be female and SBO patients, and had longer times from symptom onset to surgical intervention and length of hospital stay (HLOS) (Table 2; PAS versus naïve; median ages 59 years old versus 31, P<0.001; 86.7% female versus 35.9%; P = 0.002; 60% SBO versus 2.6%, P<0.001; 84.7h from symptom onset to surgery versus 22.3h, P<0.001; median HLOS 7 days versus 2, P<0.001, S3 Table). For appy patients only, Alvarado scores were calculated as a prediction of the diagnosis of appendicitis and assigned to categories based on score [45]. We found no difference in Alvarado score severity between PAS- and naïve-appy patients (P = 0.517). Of comorbidities, PAS patients had higher rates of hypertension, hypothyroidism, and of patients with a history of abdominal disease, PAS patients were more likely to have suffered a previous SBO (46.7 versus 12.8% hypertension, P = 0.01; 40 versus 2.6% hypothyroidism, P = 0.001; 20 versus 0% prior SBO, P = 0.01). The differences in these clinical parameters and the observation that more PAS patients use lipid regulators (26.7 versus 5.1%, P = 0.04) are likely related to the increase in median age amongst the PAS patients. Pre-surgical administration and dosage of anti-inflammatory and anti-coagulants were collected and showed no differences between PAS versus naïve for anti-inflammatory drugs. However, 26.7% of PAS patients received the anti-coagulant Enoxaparin versus none of the naïve patients (P = 0.004), and in addition, PAS patients were administered significantly more fluid volume (median 4500 versus 1500 ml, P = 0.001) these findings may be related to the increased time prior to procedure and the hospital length of stay of PAS patients.

**Table 2:** Clinical characteristics of the cohort of 54 patients selected for immunomodulator analysis.

	Naïve	PAS^a^	P
	n = 39 (72%)	n = 15 (28%)	
Age (median, IQR^b^)	31 (24)	59 (28)	<0.001
Sex (% female)	35.9% (14)	86.7% (13)	0.002
BMI (median, IQR)	25.5 (8.9)	27.8 (6.6)	0.866
Symptom onset to procedure (median hours (IQR))	22.3 (17.9)	84.7 (48.0)	<0.001
Hospital length of stay (median days (IQR))	2 (1)	7 (7)	<0.001
% SBO patients	2.6% (1)	60% (9)	<0.001
Alvarado Category (APPY patients only, range of scores)			0.517
Low (2–4)	23.7% (9)	16.7% (1)	
Moderate (5–6)	23.7% (9)	50% (3)	
High (7–9)	52.6% (20)	33.3% (2)	
Comorbidities			
Diabetes	5.1% (2)	13.3% (2)	0.31
Hypertension	12.8% (5)	46.7% (7)	0.01
Hyperlipidemia	10.3% (4)	33.3% (5)	0.1
Renal Disorder	2.6% (1)	13.3% (2)	0.18
GERD	12.8% (5)	28.7% (4)	0.24
Hypothyroidism	2.6% (1)	40.0% (6)	0.001
History of abdominal disease	2.6% (1)	33.3% (5)	0.01
Colonic Diverticulosis	0% (0)	6.7% (1)	0.28
Chronic Gastritis	0% (0)	6.7% (1)	0.28
Gallstone Pancreatitis	0% (0)	6.7% (1)	0.28
Colitis	2.6% (1)	0% (0)	>0.99
Peritonitis	0% (0)	6.7% (1)	0.28
Ovarian cyst rupture	0% (0)	6.7% (1)	0.28
Prior appendectomy	0% (0)	6.7% (1)	0.28
Prior SBO	0% (0)	20.0% (3)	0.01
Hernia	5.1% (2)	20.0% (3)	0.12
Number of prior abdominal surgeries			<0.001
0	100% (39)	0% (0)	
1	0% (0)	40% (6)	
2	0% (0)	33.3% (5)	
3	0% (0)	6.7% (1)	
4	0% (0)	13.3% (2)	
7	0% (0)	6.7% (1)	
Pre-Admission Medications			
Anti-Inflammatory	10.3% (4)	26.7% (4)	0.197
Lipid Regulators	5.1% (2)	26.7% (4)	0.044
Anti-Thrombotic	0% (0)	6.7% (1)	0.278
Drug Administration and Total Volume (median (IQR))			
Dexamethasone	69.2% (27)	60.0% (9)	0.52
Dexamethasone Volume	4 (4)	4 (4)	0.8
Ketorolac	18.0% (7)	26.7% (4)	0.48
Ketorolac Volume	15, (0)	52.5 (60)	0.22
Enoxaparin	0% (0)	26.7% (4)	0.004
Enoxaparin Volume	N/A	40 (0)	N/A
Heparin	0% (0)	6.7% (1)	0.27
Heparin Volume	N/A	81800	N/A
IV Fluid Administration and Volume (median mL (IQR))			
Any fluid volume	1500 (1250)	4500 (3100)	0.001
Normal saline	69.2% (27)	80.0% (12)	0.52
Total saline volume	1000 (1000)	1000 (500)	0.72
Lactated ringers	76.9% (30)	80.0% (12)	>0.99
Total lactated ringer’s volume	1000 (500)	1000 (2500)	0.08
D5 12 NS with 20 KCl^c^	0% (0)	6.7% (1)	0.28
Total D5 12 NS with 20 KCl volume	N/A	14000 (0)	N/A
D5W^d^	0% (0)	6.7% (1)	0.28
Total D5W volume	N/A	3100 (0)	N/A

Decision points illustrated in Fig 2A resulted in 54 PAS and naïve patient samples selected for further analysis. Clinical characteristics were compared between 39 naïve and 15 PAS patients; P<0.05 was considered significant.

^a^ Previous abdominal surgery

^b^ Interquartile range

^c^ Potassium Chloride in Dextrose and Sodium Chloride

^d^ Dextrose 5% in water

Naïve patients had increased white blood cell counts (13.8 x 10^3^ cells/μL versus 9.9, P<0.01, Table 3) implying increased systemic inflammation likely relating to the proportion of appy patients. PAS patients had lower hemoglobin (13 g/dL versus 15.8, P<0.01), hematocrit (40.1% versus 45.8%, P<0.01), and fewer red blood cells (4.3 x 10^6^ cells/μL versus 5.1 x 10^6^, P<0.01) likely related to age and sex differences between the two groups. PAS patients showed decreased mean corpuscular hemoglobin concentration (32.9 g/dL versus 34.3, P<0.01) and increased blood chloride (107 mm/L versus 106, P = 0.039). No other measured chemistry values were significantly different between naïve and PAS patients.

**Table 3:** Lab and chemistry values of the cohort of 54 patients selected for immunomodulator analysis.

	Naïve	PAS	P
	n = 39 (72%)	n = 15 (28%)	
Lab values			
White blood cell count	13.8 (4.3)	9.9 (3.5)	0.001
Red blood cell count	5.1 (0.65)	4.3 (0.68)	<0.001
Hemoglobin	15.8 (2.3)	13 (1.8)	<0.001
Hematocrit %	45.8 (4.9)	40.1 (4.7)	<0.001
Platelet count	255 (60.5)	266 (64.5)	0.346
Mean corpuscular volume	88 (5)	93 (4.3)	0.135
Mean corpuscular hemoglobin	30.4 (1.3)	30.9 (2.7)	0.559
Mean corpuscular hemoglobin concentration	34.3 (1.5)	32.9 (2)	<0.001
Chemistry values			
Sodium	138 (3.5)	138 (4)	0.256
Potassium	3.7 (0.3)	3.7 (0.45)	1
Chloride	106 (4)	107 (6.5)	0.039
Carbon dioxide	24 (3)	23 (3.5)	0.505
Anion gap	12 (4)	10 (3)	0.984
Blood urea nitrogen	13 (4.5)	19 (10)	0.464
Serum creatinine	1 (0.24)	0.87 (0.3)	0.098
Glucose	106 (23)	107 (20)	0.634

Decision points illustrated in Fig 2A resulted in 54 PAS and naïve patient samples selected for further analysis. Lab chemistry values were compared between 39 naïve and 15 PAS patients; P<0.05 was considered significant.

#### Reactive ascites collected from patients with a history of PAS had lower levels of seven immunomodulators and higher levels of two c-c-motif chemokines

Mesothelial, fibroblasts, peritoneal macrophages, and other immune cells regulate adhesion formation and immune cell recruitment by releasing immunomodulators into the peritoneal cavity resulting in reactive ascitic fluid accumulation [25]. We hypothesized that patient rA samples containing such molecules likely contribute to the unique phenotypes observed in rA-treated MCs; thus, we quantified 85 immunomodulating proteins in our cohort of patient rA (Fig 2A). A total of 79 proteins met criteria for further analysis, and we first performed a univariate analysis comparing PAS versus naïve rA, and found 9 proteins that differed between the groups, 7 of which were less abundant in PAS patient rA (Fig 2B, fold-regulation > |2|, FDR<0.05; Tables 4, S4 and S5). Interleukin-1 receptor antagonist (IL-1RA; -50.82-fold), leukemia inhibitory factor (LIF; -6.86-fold), IL-10 (IL-10; -5.12-fold), granulocyte-colony stimulating factor (G-CSF; -10.14-fold, FDR<0.001), Interferon (IFN)-γ inducible protein 10 (IP-10/CXCL10; -9.7-fold), soluble vascular endothelial growth factor receptor-1 (sVEGFR1; -11.96-fold), and Eotaxin-2 (-3.18-fold) showed lower concentrations in PAS versus naïve patient rA. Conversely, two C-C-motif chemokines were more abundant in PAS versus naïve rA, CCL21, also known as 6CKine (2.64-fold) and C-C motif chemokine ligand 27 (CCL27), also known as, cutaneous T cell-attracting chemokine (CTACK; 2.07-fold). Within this group of cytokines, the top 3 most concentrated immunomodulators (median (IQR) measured in rA collected from surgically naïve patients were IP-10 (2207.57 pg/ml (4859)), sVEGFR1 (1991.27 pg/ml (2074)), and G-CSF (1418.96 pg/ml (1561)) (Table 4). In PAS patient rA, IP-10 (227.47 pg/ml (365)), 6CKine (320.04 pg/ml (280)), and CTACK (311.16 pg/ml (152)) were the top 3 most abundant amongst these proteins (Tables 4 and S5).

**Table 4:** Differentially abundant immunomodulators between PAS and naïve patient samples.

Analyte	Gene	Name	Naïve	PAS	Fold-regulation	Log2 Fold-change	FDR adj P	-Log_10_ (adj P)
			n = 39 (72%)	n = 15 (28%)				
IL-1RA	IL1RN	interleukin 1 receptor antagonist	272.9^a^ (47–885)	5.37^a^ (2–153)	-50.82	-5.67	1.95E-02	1.71
LIF	LIF	LIF interleukin 6 family cytokine	51.75 (18–180)	7.54 (3–28)	-6.86	-2.78	2.24E-02	1.65
IL-10	IL10	interleukin 10	377.81 (127–1078)	73.78 (30–107)	-5.12	-2.36	2.35E-03	2.63
G-CSF	CSF3	colony stimulating factor 3	1418.96 (862–2423)	139.92 (21–684)	-10.14	-3.34	2.35E-03	2.63
IP-10	CXCL10	C-X-C motif chemokine ligand 10	2207.57 (647–5506)	227.47 (117–482)	-9.70	-3.28	1.06E-02	1.98
sVEGFR1	FLT1	fms related tyrosine kinase 1	1991.27 (666–2740)	166.49 (113–754)	-11.96	-3.58	5.10E-03	2.29
Eotaxin-2	CCL24	C-C motif chemokine ligand 24	277.1 (191–575)	87.02 (53–149)	-3.18	-1.67	6.00E-04	3.22
6CKine	CCL21	C-C motif chemokine ligand 21	121.44 (71–232)	320.04 (138–418)	2.64	1.40	2.83E-02	1.55
CTACK	CCL27	C-C motif chemokine ligand 27	149.99 (110–193)	311.16 (202–354)	2.07	1.05	4.90E-03	2.31

rA collected from 39 naïve and 15 PAS patients were subjected to quantification of 85 immunomodulator protein. Analyte concentrations for naïve versus PAS patient samples were compared and an FDR adjusted P<0.05 was considered significant.

^a^ Values are displayed as median picograms/milliliter (quartile 1- quartile 3).

Shown in Fig 2C is a heatmap depicting the relative concentrations of these 9 immunomodulators in each individual patient rA parsed by PAS/naïve and FIB-GCX groups. The concentrations of the PAS rA sample proteins showed a striking consistency within the samples that formed highFIB-highGCX (arrowed bracket; Fig 2C).

Likely because of the low number of PAS rA samples, two of the fiber-GCX phenotypes were not observed when MCs were treated with rA collected from PAS patients (Fig 2A). Thus, two crosswise comparison groups would be missing in a statistical multivariate analysis of PAS-FIB-GCX groups. Due to these constraints, we combined the PAS and FIB-GCX variables to form 6 comparison groups and performed a univariate analysis to detect immunomodulator differential abundance between groups (S4, S6 and S7 Tables).

#### PAS/naïve-FIB-GCX comparison cohorts further delineate differentially abundant immunomodulators

**Fig 3:**
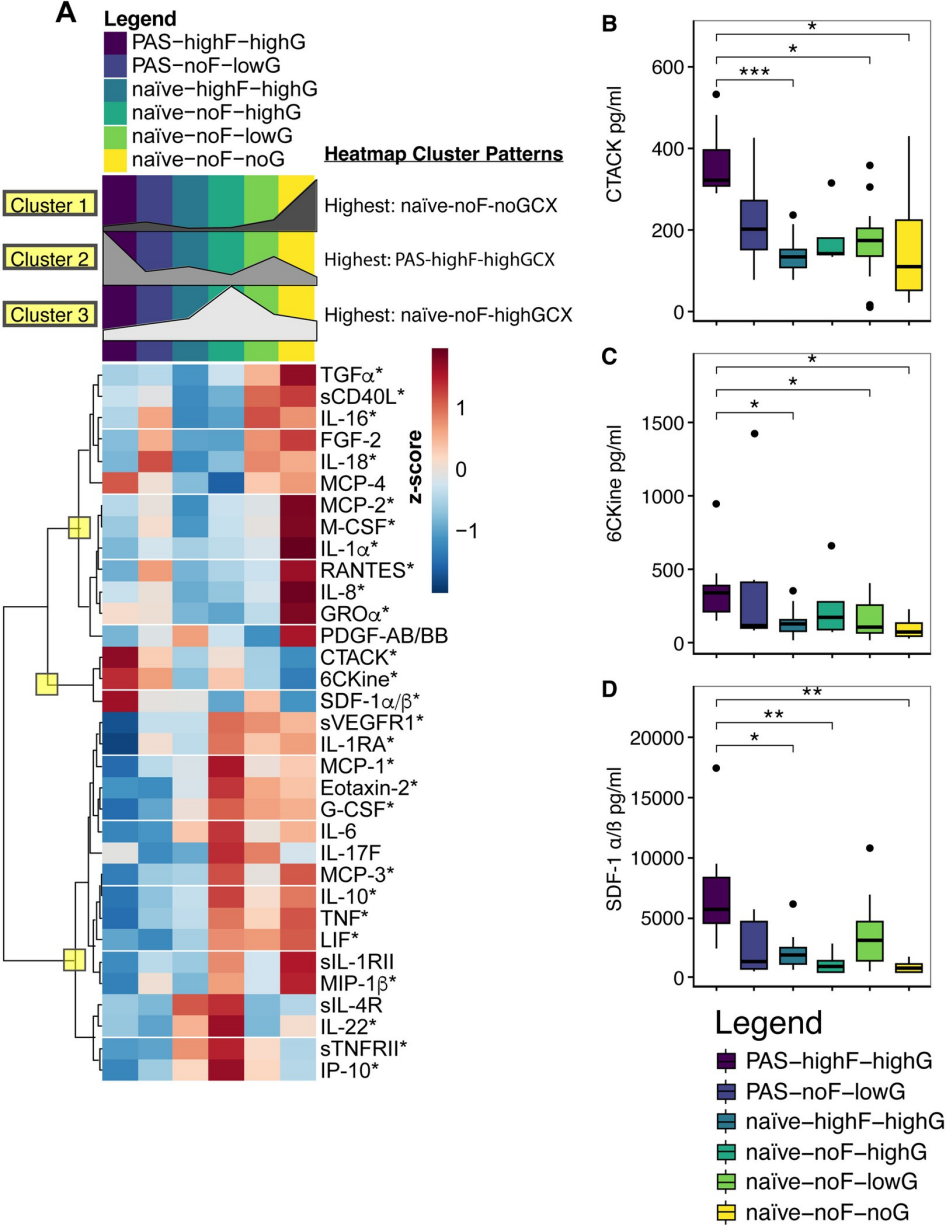
Immunomodulators differ between PAS-Fiber-GCX groups. (A) Heatmap showing 33 immunomodulators that significantly differed in PAS-Fiber-GCX samples (Kruskal Wallis; P<0.05). Asterisks mark 26 proteins that have at least one significant Dunn’s pairwise comparison between PAS-Fiber-GCX groups. Yellow boxes delineate nodes of hierarchical clustering that separated three major heatmap cluster patterns. Heatmap cluster patterns as calculated using the sum of the median protein concentrations within each hierarchical cluster for each separate PAS-Fiber-GCX group. (B–D) Boxplots of the IQR and median pg/ml (horizontal line) of three proteins significantly more abundant in PAS-highFIB-highGCX. (B) Boxplot graph depicting the median (IQR) of CTACK, which is 1 of 4 (CTACK, IL-1RA, IP-10, and sCD40L) immunomodulators that show the most significant number of pairwise comparisons between groups. (B and C) CTACK and 6CKine are significantly more abundant in PAS-highFIB-highGCX compared to naïve-highFIB-highGCX, naïve-noFIB-lowGCX, and naïve-noFIB-noGCX groups. (D) In PAS-highFIB-highGCX, SDF-1 is more abundant compared to naïve-highFIB-highGCX, naïve-noFIB-highGCX, and naïve-noFIB-noGCX. FDR-adjusted q-value symbols: *** FDR<0.001; ** FDR<0.01; * FDR≤0.05).

Immunomodulator abundance of the six cohorts of rA samples defined by PAS-FIB-GCX were compared by Kruskal Wallis, and out of 79 proteins, 33 were found to be significantly different between groups (Fig 3A). Based on hierarchical clustering nodes (Fig 3A; yellow boxes), we calculated heatmap cluster patterns based on the sum of the median concentrations of the proteins within each of the three main clusters (Fig 3A and S6 Table). Cluster 1 showed higher protein concentrations in the naïve-noFIB-noGCX group, cluster 2 proteins were the most concentrated in PAS-highFIB-highGCX samples, and cluster 3 was defined by higher protein concentrations in naïve-noFIB-highGCX samples. Dunn’s pairwise comparisons (pwc) of PAS-FIB-GCX groups showed that 26 proteins had at least one significant pwc after FDR P-value adjustment (asterisks, Fig 3A and S7 Table).

To gain a global understanding of the similarities or dissimilarities between PAS-FIB-GCX groups regarding these immunomodulators, we calculated the number of significant pwc between each of the 15 possible comparison groups, and found a total of 77 pwc were found to be significant (Table 5). The two groups that were found to be significantly different over 13 immunomodulators, 17% of the findings, were PAS-highFIB-highGCX versus naïve-noFIB-highGCX. Notably, PAS-highFIB-highGCX samples occupied the 3 topmost significant pwc, and overall represented 49% of the total significant pairwise comparisons implying that these samples are vastly different from most of the other sample groups. Remarkably, the top 6 comparison groups with the greatest number of significant pwc all shared in the contrast of high versus no fiber formation, regardless of PAS vs naïve or the relative level of GCX production between the groups. These results imply that the immunomodulators that initiate the formation of fibers by the MCs are strong drivers of the differences between these groups.

**Table 5:** Overview of the Dunn’s pairwise comparison results sorted by number of significant findings.

Pairwise comparison 1^st^ group	Pairwise comparison 2^nd^ group	# of FDR≤0.05	%
PAS-highFIB-highGCX	Naïve-noFIB-highGCX	13	17%
PAS-highFIB-highGCX	Naïve-noFIB-noGCX	10	13%
PAS-highFIB-highGCX	Naïve-noFIB-lowGCX	8	10%
Naïve -highFIB-highGCX	Naïve-noFIB-noGCX	8	10%
Naïve-highFIB-highGCX	Naïve-noFIB-highGCX	6	8%
Naïve-highFIB-highGCX	Naïve-noFIB-lowGCX	6	8%
PAS-noFIB-lowGCX	Naïve-noFIB-highGCX	5	6%
PAS-highFIB-highGCX	Naïve-highFIB-highGCX	5	6%
Naïve-noFIB-highGCX	Naïve-noFIB-noGCX	5	6%
PAS-noFIB-lowGCX	Naïve-highFIB-highGCX	3	4%
PAS-noFIB-lowGCX	Naïve-noFIB-lowGCX	2	3%
PAS-highFIB-highGCX	PAS-noFIB-lowGCX	2	3%
Naïve-noFIB-highGCX	Naïve-noFIB-lowGCX	2	3%
PAS-noFIB-lowGCX	Naïve-noFIB-noGCX	1	1%
Naïve-noFIB-lowGCX	Naïve-noFIB-noGCX	1	1%
Total number of significant Dunn’s pairwise comparisons		77	100%

Table showing the number of significant (FDR adjusted P<0.05) pwc between all possible groups, and the percentage of the total number of significant pwc between all groups.

CTACK, 6CKine and SDF-1α/β showed significant differences in concentration between PAS-highFIB-highGCX and several of the naïve-FIB-GCX groups. CTACK and 6CKine were significantly more abundant in PAS-highFIB-highGCX (median, (IQR); CTACK: 323.62 pg/ml (87.87); 6CKine: 342.52 pg/ml (178.50)) compared to naïve-highFIB-highGCX (133.62 pg/ml (45.17); 127.59 pg/ml (82.38)), naïve-noFIB-lowGCX (175.77 pg/ml (67.44); 109.35 pg/ml (186.48)), and naïve-noFIB-noGCX groups (111.71 pg/ml (172.36); 75.45 pg/ml (86.25); Fig 3B and 3C and S6 and S7 Tables). SDF-1α/β was more concentrated in PAS-highFIB-highGCX (5654.18 pg/ml (3774.07)) compared to naïve-highFIB-highGCX (1896.90 pg/ml (1402.94)), naïve-noFIB-highGCX (903.26 pg/ml (975.76)), and naïve-noFIB-noGCX (777.30 pg/ml 684.80); Fig 3D and S6 and S7 Tables).

**Fig 4:**
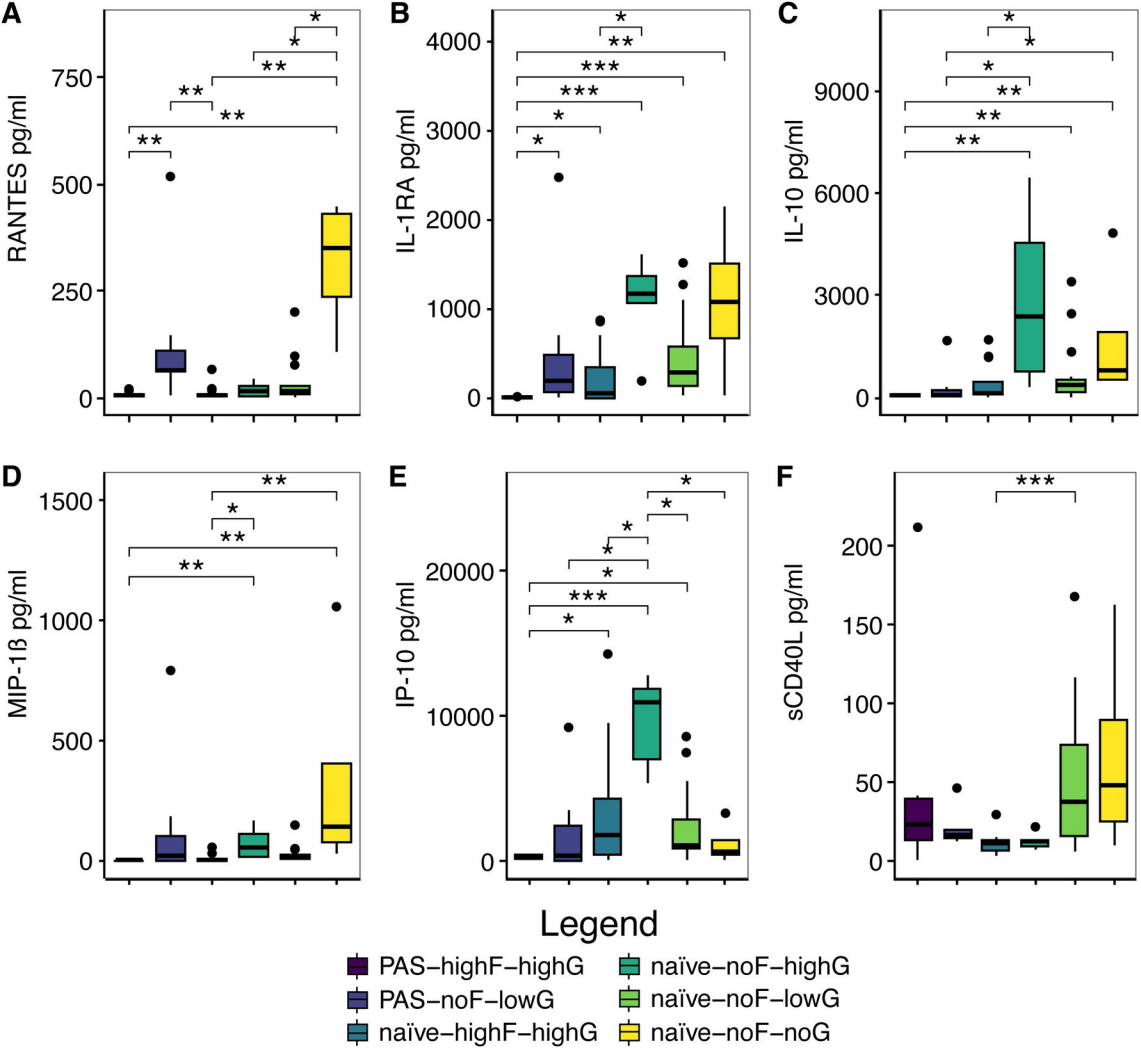
Immunomodulators with the topmost significant differences between PAS-Fiber-GCX groups. (A-E) Boxplot graphs depicting the median (IQR) of immunomodulators that comprise the topmost number of significant pairwise comparisons between groups. (A-C) RANTES, IL-1RA, and IL-10, each show 6 significant group-to-group comparisons. (B, C) PAS-highFIB-highGCX vs naïve-noFIB-highGCX, naïve-noFIB-noGCX, and naïve-noFIB-lowGCX are the 3 group-to-group comparisons with the topmost number of significantly differently abundant immunomodulators and have two proteins in common between them: (B) IL-1RA and (C) IL-10 Amongst the comparisons between naïve-highFIB-highGCX vs naïve-noFIB-noGCX, naïve-noFIB-highGCX, and naïve-noFIB-lowGCX, one protein was in common: (D) MIP-1β. (B, E, F) Out of 4 (CTACK, IL-1RA, IP-10, and sCD40L), 3 immunomodulators that show the most significant (lowest FDR) pairwise comparisons between groups, (B) IL-1RA, (E) IP-10, and (F) sCD40L. FDR-adjusted q-value symbols: *** FDR<0.001; ** FDR<0.01; * FDR≤0.05.

PAS-highFIB-highGCX versus PAS-noFIB-lowGCX showed differential protein abundance in 2 analytes: RANTES (6.52 pg/ml (4.51) vs 65.09 pg/ml (48.68); FDR<0.01; Fig 4A) and IL-1RA (median, (IQR); 2.25 pg/ml (2.36) vs 188.13 pg/ml (410.40); FDR<0.05; Fig 4B). Amongst the 3 comparison groups with the highest number of significant pwc, PAS-highFIB-highGCX vs naïve-noFIB-highGCX, naïve-noFIB-noGCX, and naïve-noFIB-lowGCX, two analytes were in common: IL-1RA and IL-10 (Table 5 and Fig 4B and 4C). Amongst the comparisons between naïve-highFIB-highGCX vs naïve-noFIB-noGCX, naïve-noFIB-highGCX, and naïve-noFIB-lowGCX, one analyte was in common: MIP-1β (Table 5 and Fig 4D). IP-10 group comparisons most closely resembled cluster 3 (Fig 3A) where the highest relative concentration of IP-10 was in naïve-noFIB-highGCX rA patient samples. Four immunomodulators, CTACK, IL-1RA, IP-10, and sCD40L, showed the most significant (lowest FDR) pairwise comparisons between groups (Figs 3A, 4B, 4E and 4F).

### Ingenuity Pathway Analysis (IPA) of differentially abundant immunomodulators between PAS-Fiber-GCX groups

The rA fluids in this study were collected, without dilution with lavage fluid, immediately prior to surgical intervention and represent a snapshot of signaling pathways that were active at the time of collection. Identification of active signaling may provide insight into mechanisms that contributed to the distinct PAS-Fiber-GCX phenotypes. To identify and characterize potential signaling pathways, we leveraged Ingenuity Pathway Analysis (IPA), a database of curated knowledge from published literature. IPA “signaling pathways” provide a visual overview of complex biological processes, and additionally, the software predicts activation or inhibition of signaling pathways based on literature findings.

#### PAS patient rA that elicited high fiber and high GCX formation in mesothelial cells show predicted inhibition of pro- and anti-inflammatory pathways

**Fig 5:**
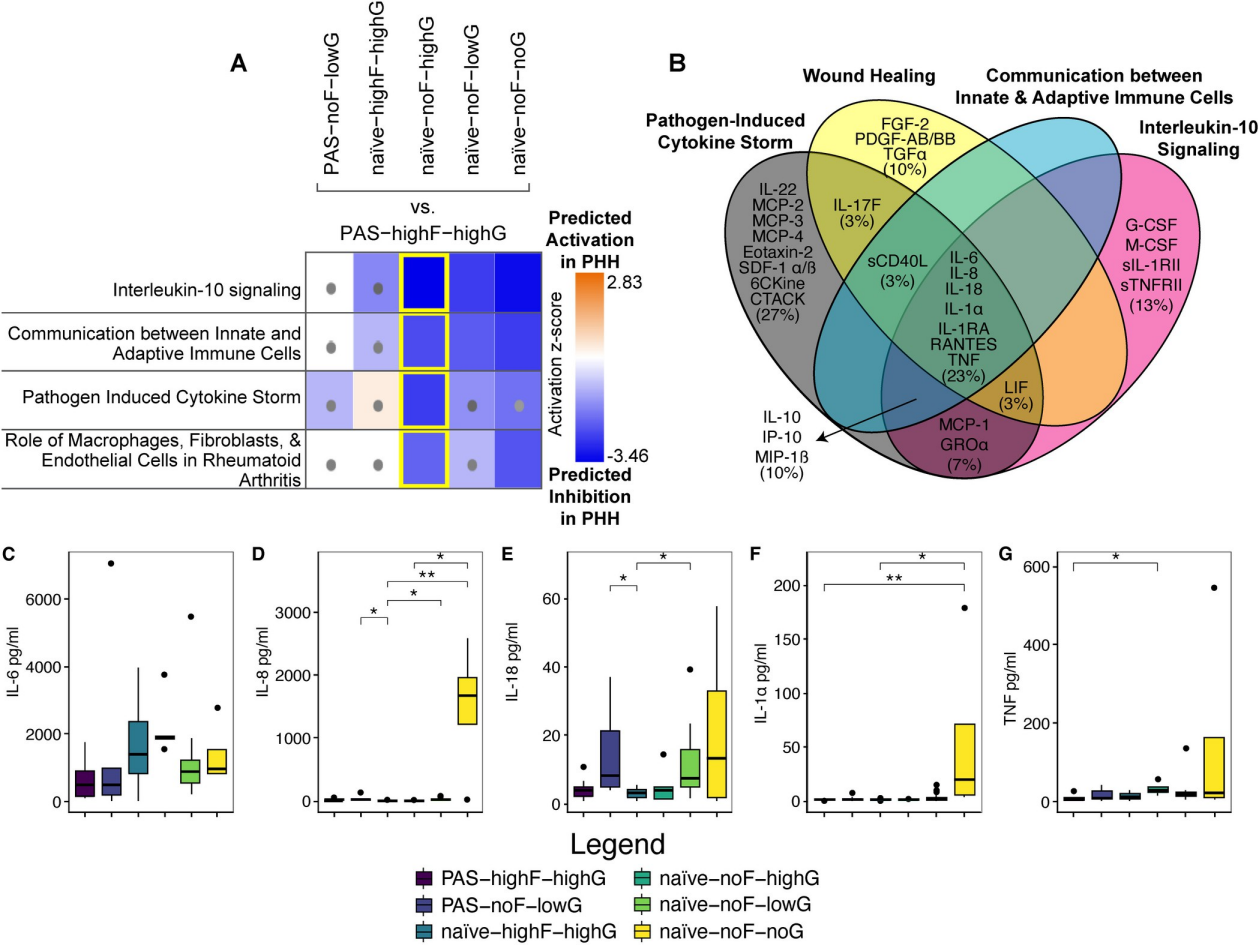
Pathway analysis shows predicted inhibition of pro- and anti-inflammatory pathways in PAS-highFIB-highGCX when compared to naïve-noFIB-GCX samples. (A) Heatmap of “activation z-scores” for the pathways with the strongest predictions based on the directionality of the molecules within the comparison groups. Signaling pathways with activation z-scores of < -2 (purple) are predicted to be inactive or suppressed; activation z-scores of > 2 (orange) are predicted to be activated; squares with grey dots mark pathways with weak prediction z-scores between -2 and 2. Yellow boxes highlight PAS-highFIB-highGCX versus naïve-noFIB-highGCX, which had 4 predictions with activation z-scores < -2. (B) Four-way Venn diagram depicting the representation of immunomodulators found to be differentially abundant between PAS-Fiber-GCX groups in the following IPA signaling pathways: “Pathogen-Induced Cytokine Storm” (black oval), “Wound Healing” (yellow oval), “Communication between Innate and Adaptive Immune Cells” (cyan oval), and Interleukin-10 signaling (magenta oval). (C-G) Boxplots depicting the median pg/ml (IQR) of IL-6 (C), IL-8 (D), IL-18 (E), IL-1α (F), and TNF (G). FDR-adjusted q-value symbols: *** FDR<0.001; ** FDR<0.01; * FDR≤0.05. Note: Kruskal-Wallis statistical analysis of IL-6 showed an unadjusted P = 0.031; however, none of the Dunn’s pwc FDR adjusted q values were <0.05).

For IPA analysis, we calculated log_2_ fold-changes comparing the PAS/naïve-Fiber-GCX groups back to PAS-highFIB-highGCX and performed a “Core Analysis” of immunomodulators (S8 Table) that were significantly more than 2-fold differentially abundant between comparison groups (Dunn’s pwc; FDR<0.05; Figs 5A and S4). Based on the directionality and significance of the log_2_ fold-change values in each comparison dataset, IPA predicted that “Interleukin-10 signaling” (S5 Fig), “Communication between Innate and Adaptive Immune Cells” (S6 Fig), “Pathogen Induced Cytokine Storm Signaling Pathway” (S7 Fig), and “Role of Macrophages, Fibroblasts, and Endothelial Cells in Rheumatoid Arthritis” (S8 Fig) showed the strongest projected “activation z-scores.” These pathways showed activation z-scores of less than -2 in at least one comparison dataset, indicating a predicted inactivation or suppression of the pathway by molecules in PAS-highFIB-highGCX samples compared to the other groups (Fig 5A). Pathway-comparison dataset squares with grey dots showed weak predictions with z-scores between -2 and 2 indicating that the data in these comparisons did not have enough predictive value, which may happen when the directionality of molecules are inconsistent within the pathway or that there are too few molecules that match to the pathway to yield a strong prediction (Figs 5A and S4).

The dataset comparing immunomodulators in PAS-highFIB-highGCX versus naïve-noFIB-highGCX rA showed the most consistent and significant activation z-scores for 11 of the top 15 pathways predicted to be associated with these data (yellow boxes, Figs 5A and S4). When compared to naïve-noFIB-highGCX and naïve-noFIB-noGCX, the one pathway that was predicted to be activated by signaling molecules present in PAS-highFib-highGCX was LXR/RXR activation (S4 Fig). Apart from LXR/RXR activation, all other pathways with significant z-score values were predicted to be inhibited based on PAS-highFIB-highGCX analyte levels versus the other naïve rA fluids that did not induce FIB formation (naïve-noFIB; S4A Fig). These data provide further support that FIB formation is a major driver of the differences in analyte abundance between the groups.

To better understand connections between differentially abundant immunomodulators and IPA signaling pathways with respect to adhesion formation, we identified proteins in common between “Interleukin-10 Signaling,” “Pathogen-Induced Cytokine Storm”, “Communication between Innate and Adaptive Immune Cells”, and “Wound Healing” (Figs 5B, S5–S7 and S9). Of the 33 analytes that were significantly differentially abundant by Kruskal-Wallis testing (P<0.05), 23, 13, 11, and 17 proteins matched to the following pathways: “Pathogen-Induced Cytokine Storm” (black oval), “Wound Healing” (yellow oval), “Communication between Innate and Adaptive Immune Cells” (cyan oval), and Interleukin-10 signaling (magenta oval) (Fig 5B). Seven immunomodulators were found to be in common between all the 4 datasets: RANTES, IL-1RA, IL-6, IL-8, IL-18, IL-1α, and TNF (Figs 4A, 4B and 5C through 5G).

## Discussion

### Summary of findings

Herein, we analyzed an expanded number of appy and SBO patient rA samples where quantified immunomodulators were compared between patients with a history of PAS (PAS rA) or with surgically naïve abdomens (naïve rA). Importantly, patient fluids were collected without lavage prior to surgical intervention for appy or SBO and provide a snapshot of localized proteins representing the individualized immune response of each patient.

Furthermore, culturing MCs with patient rA resulted in prominent changes related to cell shape and behavior. We observed that select rA initiated MCs to generate a layer of GCX that, in some cases, also resulted in the formation of dense thread-like ECM fibers. Further, we observed that other rA caused MCs to aggregate into star-like clusters that connected via tracks of aligned fibroblast-like cells without fibers or GCX. Based on scoring of MC morphologies, we designated each patient rA into five distinct “fiber-GCX” (FIB-GCX) groups: highFIB-highGCX, highFIB-lowGCX, noFIB-highGCX, noFIB-lowGCX, and noFIB-noGCX groups. These groups were further divided based on whether the rA was collected from PAS or naïve patients. Differential analysis of immunomodulator abundance between PAS-FIB-GCX groups showed an overall muted immune response by PAS patients regardless of FIB-GCX abundance, implying a suppressed or insufficient immune response to appendicitis or adhesive SBO.

Initially, we hypothesized that rA samples that induced dense thread-like ECM fibers would show high levels of pro-adhesion and pro-inflammatory proteins. However, the highest levels of pro-inflammatory and pro-adhesion proteins were found in naïve rA fluids that did not induce ECM fibers or GCX secretion by MCs.

### Patient rA fluids stimulate resting MCs to form GCX and/or ECM fibers, while other rA fluids result in fibroblastic mesothelial morphology

We previously published that reactive ascitic peritoneal fluid collected from APPY and SBO patients may provide a model system for discovery of the mechanisms leading to abdominal adhesion formation. Appendicitis arises from overgrowth of bacteria in the appendix, and as a result, the body attempts to isolate the infection from the rest of the body and may build a fibrous ‘cocoon’ around the inflamed appendix [46]. Inflammation activated by bacterial PAMPS increases vascular permeability allowing for reactive ascitic exudate to accumulate in the peritoneal cavity [6]. Adhesive SBO may cause localized hypoxia leading to tissue necrosis and PAMP/DAMP activation of inflammation [6, 47].

Under resting non-inflammatory conditions, the scant peritoneal fluid that is present contains tissue plasminogen activator (tPA) that actively inhibits fibrin matrix assembly, the first phase of adhesion formation [25]. Upon activation, MCs express plasminogen activator inhibitors (PAI), decreasing fibrinolysis and allowing for fibrin matrix assembly. Chronic fibrous adhesions are hypothesized to be maintained by active inflammation leading to an imbalance between fibrin matrix deposition and fibrinolysis [11, 13–15]. Another mechanism besides fibrinolysis that insures a non-adhesive surface of the peritoneum under resting conditions is MC production of pericellular GCX [48]. Further, MC pericellular GCX is disrupted by injury to the membrane and results in upregulation of GCX components expressed by MCs [49]. In vivo, GCX and fibrin matrix assembly are MC cellular responses to injury and inflammation; thus, we rationalized that the cellular responses to rA collected from appendicitis or adhesive SBO patients may be recapitulated by treating resting primary human MCs in vitro with neat patient rA fluid.

Resting MCs rapidly responded to in vitro exposure of patient rA and within hours initiated major morphological transformations. Select rA drove GCX production, and MCs stimulated with select rA fluids produced dense GCX impenetrable to phalloidin. To allow phalloidin access to the f-actin cytoskeleton, the GCX of treated MCs was digested by hyaluronidase. These observations support the hypothesis that a major component of the GCX induced by select patient rA is hyaluronan [5]. Several cell types, including MCs, are known to have high expression of endogenous hyaluronan, a glycosaminoglycan made of N-acetylglucosamine and glucuronic acid subunits located in GCX [5]. We discovered that GCX produced by rA-stimulated MCs stained with WGA, a lectin known to bind to N-acetylglucosamine and sialic acid and ConA, a lectin that binds to α-D-mannosyl and α-D-glucosyl residues in GCX [43, 50]. When peritoneum cell surface GCX is damaged, the function of MCs as a regulators of fluid transport is disrupted, as is the case during surgery and inflammation.

Adhesions originate as a matrix of fibrin that forms following MC inhibition of fibrinolysis, and when treated with select patient rA, a dense layer of ECM fibers, as visualized under brightfield microscopy, formed on top of the MCs. These ECM fibers may be comprised of fibrin and resemble peritoneum fibrin deposition that is capable of mediating metastatic cancer adhesion [51].

Upon activation MCs are known to undergo a mesothelial to mesenchymal transition that results in a pro-fibrotic phenotype [6]. We observed that rA treated MCs showed fibroblastic; and further, some rA fluids resulted in MCs forming star-like clusters of cells that were connected via intercalated cells, much like a zipper.

Our observed responses of rA-treated MCs resembled established responses of MCs when regulating of adhesion formation. These cellular responses are likely dictated by what proteins and molecules are locally present; thus, we quantified immunomodulators in rA collected from appy patients experiencing infectious inflammation and patients undergoing adhesiolysis to treat SBO.

### Immunomodulators quantified in patient rA provide insight into possible mechanisms of inflammatory-mediated and post-surgical adhesion regulation

Inflammatory mediators are secreted into rA by free-floating peritoneal macrophages, mesothelial cells, and resident fibroblasts in the early response to infection and injury [7–10, 52, 53]. Further, inflammation increases vascular permeability, and the rA fluids in this study are exudates that contain plasma proteins [26]. Plasma reference intervals for the HD71 multi-plex assay used in this study have been estimated by Eve Diagnostics (Calgary, AB Canada) through data mining of >9000 plasma samples collected from healthy and pathological samples. To contextualize our results, we compared the median concentration of analytes from each PAS-FIB-GCX group and found that across all groups, IL-6, IL-10, and G-CSF were above the max reference interval for plasma. IL-6 and IL-10 are established mediators of abdominal adhesion [25]. G-CSF is a neutrophil chemoattractant and inhibition of neutrophils decreases induction of adhesions [54]. Despite these analytes having higher concentrations in patient rA samples compared to circulating plasma, in the comparison of PAS versus naïve rA samples, PAS rA showed significantly lower levels of IL-10 and G-CSF. Moreover, IL-6 was trending lower in PAS versus naïve rA (median (IQR) 502.01 (787) pg/ml vs 1139.3 (1158) pg/ml; q-value = 0.055).

IL-1 signaling plays a critical role in adhesion formation and immune cell recruitment, and IL-1α is necessary for the recruitment of neutrophils to the peritoneal cavity through induction of GROα, a neutrophil chemoattractant, and is released by mesothelial cells [55–57]. The highest IL-1α was observed in naïve-noFIB-noGCX samples that did not initiate high levels of GCX nor ECM fiber formation. In a mouse adhesion model, IFN-γ, IL-6, TNF, and transforming growth factor (TGF) β, a cytokine that is fundamental to fibrosis and peritoneal adhesions, increased 3h post-injury in lesions along with an infiltration of neutrophils [54, 58, 59]. IL-6 and IL-1α, and GROα-mediated neutrophil infiltration drive abdominal adhesion formation [60, 61], and these proteins were more concentrated in naïve-noFIB-noGCX. Further, intra- and extracellular IL-1 signaling is actively inhibited by IL-1RA and acts to balance IL-1 signaling [62]. Polymorphisms of IL-1RA, resulting in decreased IL-1RA expression, are associated with an increased risk of abdominal adhesion formation in women with adhesions [63]. Interestingly, the lowest IL-1RA levels were found in highFIB-highGCX inducing samples that had been collected from PAS patients, likely with post-surgical abdominal adhesions.

Based on the prevailing concept of chronic inflammation and adhesion remodeling in pathogenic adhesion formation, we initially postulated that PAS rA-samples that induced MCs to produce ECM fibers would be associated with higher levels of proinflammatory, pro-adhesion, and immune chemotactic proteins. However, our analyses show that rA samples with this type of inflammatory mediator profile were collected from patients with naïve abdomens that do not appear to cause ECM fibers nor high levels of GCX secretion in MCs.

### Study limitations

This study has several limitations and may not represent the full gamut of mesothelial cell responses to rA stimulation nor all patient clinical characteristics with respect to appendicitis, SBO, or abdominal adhesion formation. The highest risk factor for abdominal adhesion formation is PAS; thus, we separated patient fluids by history of PAS. Two additional clinical risk factors for abdominal adhesion, age and sex may have confounded the PAS variable due to skewed distribution. The % female and median age variables for PAS patient rA samples at the time of this study was 86.7% female with a median age of 59 versus naïve patients that were 35.9% female with a median age of 31 (P<0.001). Furthermore, 60% of the 15 PAS patient samples were collected during surgery for SBO versus one patient (2.6%) with a naïve abdomen undergoing adhesiolysis for SBO.

We were unable to do a power calculation a priori as we would need to identify outcomes from similar studies, which do not currently exist. In consideration of the small sample size and the high number of variables in our study, we used an FDR-adjusted P-value of ≤0.05. Moreover, adjusted IPA analyses were performed by limiting the Reference Set to “User Dataset.” This helps to mitigate bias by evaluating proteins of interest (as defined by fold-change and FDR-adjusted P-value cutoffs) against only the proteins in the uploaded dataset when ranking statistical significance in the analyses.

### Concluding statement

This is an ongoing clinical study actively recruiting surgical patients, and despite limited numbers of samples, we have shown that the production, or lack of production of ECM fibers and lectin-stained GCX is significantly associated with different abundances of immunomodulators that are involved in innate immune activation, inflammation, and are known contributors to adhesion dynamics. It is currently unknown what the clinical implications of ECM fiber formation are but further investigation into whether these surface fibers support cell adhesion is warranted. As reflected by immunomodulator concentrations found in patient rA, this study captures a snapshot of signaling pathways that were active prior to surgery for non-perforated appendicitis or SBO. We show that rA from surgically naïve abdomens show strong immune responses and pro-adhesion signaling, supporting de novo adhesion formation observed by clinicians during appy. Further, our data suggest that abdominal surgery may negatively impact future immune responses to appy or SBO. Adhesions may form in response to appendicitis regardless of surgical intervention; however, both operative and non-operative management of appendicitis has negative consequences. Adhesions form in response to appendicitis prior to surgical intervention and an understanding of the similarities and differences between adhesion formation in response to inflammation versus surgical injury/wound healing is paramount. We envision a personalized medicine approach where fluid taken from abdominal surgery patients, upon peritoneal entry and before surgical intervention, would be analyzed for a broad spectrum of immunomodulators, such as in this study. The immune response expression pattern of each individual patient could be evaluated leading to a treatment plan that considers the normal healing process and pathogenic adhesion formation.

## Supporting information

S1 Fig Cuboidal-like MCs treated with patient rA increase cell-cell interactions and Alcian blue stained extracellular matrix. (A) Removing EGF from culture media drives mesothelial cells into a cuboidal-like morphology. Filamentous actin visualization with Phalloidin staining (Grayscale) on mesothelial cells in 2% media with EGF and 2% media without EGF. (B) Cartoon depicting cell-cell interaction scores corresponding to changes in MC morphology. Graph of cell-cell interaction scoring results following nested 1-way ANOVA of scoring from 3 independent experiments. Morphological changes were accessed by brightfield, and rA-treated MCs increased cell-to-cell interactions compared to controls (low interaction = 1, processes between cells = 2, dense thread-like fibers = 3, mean score ± SD: controls (2% Control and HS) 1.71 ± 0.46, appy and SBO rA-treated 2.31 ± 0.55, p < 0.05. (C) 48h-rA treated mesothelial cells were stained with Alcian blue at a pH of 2.5 to detect sulfated and carboxylated acid mucopolysaccharides and sulfated and carboxylated sialomucins. Relative Alcian blue staining was solubilized and quantified by microplate optical density (OD) at λ630nm. The two control conditions, 2% Control and HS showed very low levels of Alcian blue staining. Three of the rA showed significantly higher levels of Alcian blue staining after 48h culture compared to HS treatment controls: median optical density at 630nm ± SD: (HS, 0.0602 ± 0.005086; vs APPY 1, 0.1323 ± 0.04955 (ns); APPY 2, 0.2803 ± 0.05526 (* P<0.05); APPY 3, 1.097 ± 0.2398 (***P<0.001); APPY 4, 0.222 ± 0.2033 (ns); SBO 1, 0.8314 ± 0.2951 (**P<0.01); SBO 2, 0.1321 ± 0.04869 (ns); SBO 3, 0.07603 ± 0.01536 (ns). Results are graphed as a boxplot of median scores (interquartile range); *P<0.05; **P<0.01; ***P<0.001.
(TIF)

S2 Fig Scoring standards for 48h rA-treated MCs. (A) Brightfield micrograph showing morphological changes in 48h rA-treated MCs from two separate patient fluids. Left panel illustrates the formation of ECM fibers that form over MCs occurring under treatment conditions of select patient rA fluids. The ECM fibers mask the overall morphology of the cells in contrast to the rA-treated MCs in the right-hand panel that did not form ECM fibers. (B-E) 48h following treatment with patient rA fluids, MCs were fixed and stained with the fluorescently labeled lectins: Concanavalin A– 488 and Wheat Germ Agglutinin– 594, and DAPI. (B) rA-treatment affected the overall dispersion of cells, and under select conditions, the cells were evenly distributed (left panels). In contrast, treatment with select rA-fluids caused the cells to form extensive clustered networks of cells (right hand panels). (C and D) As cells were not permeabilized, the fluorescent-lectin staining represents extracellular glycocalyx and extracellular matrix formation. (C) Lectin-stained fibers were scored at 5 levels corresponding to relative intensity of extracellular matrix produced by the cells. (D) In some instances, the GCX separated from the side of the culture well during fixation and staining. Caret represents the edge of the GCX, and the asterisks demark cells that stayed attached to the bottom of the tissue culture well. (E) Distinct patterns of extracellular lectin staining on the surface of the cells were observed and scored. A score of 1 showed staining that excluded the nuclear region in each cell, while a score of 2 showed extracellular staining that covered the cell evenly. Select rA treatments resulted in a mix of each staining pattern and were scored with 1.5. A score of 3 indicated that individually stained cells were difficult to ascertain due to the diffuse extracellular lectin staining of the glycocalyx.
(TIF)

S3 Fig Heatmap of the z-scored relative concentrations of immunomodulators for individual patient rA parsed by PAS-Fiber-GCX groups. (A) Heatmap showing relative concentrations for 33 immunomodulators for each individual sample rA that significantly differed in PAS-Fiber-GCX samples (Kruskal Wallis; P<0.05).
(TIF)

S4 Fig Comparison of canonical pathways enriched in ‘Core” IPA analyses of log2 fold-ratio immunomodulator expression of PAS-Fiber-GCX groups compared back to PAS-highFIB-highGCX. (A) Heatmap of activation z-scores for IPA canonical pathways showing predicted activation (z-score >2; orange) or inhibition (z-score <-2; purple) of pathways based on patterns of differential abundance observed for immunomodulators compared back to PAS-highFIB-highGCX. Boxes without color or grey dots demark pathway-dataset pairs where either no relationship or a non-significant z-score was calculated for that specific canonical pathway. Each column is a pairwise comparison of cytokine abundance in PAS-noFIB-lowGCX or naïve datasets as compared back to PAS-highFIB-highGCX. The top four pathways associated with these dataset pairs are “IL-10 signaling”, “Communication between Innate and Adaptive Immune Cells”, “Pathogen Induced Cytokine Storm Signaling Pathway”, and “Role of Macrophages, Fibroblasts, and Endothelial Cells in Rheumatoid Arthritis”.
(TIF)

S5 Fig Interleukin-10 signaling pathway ‐ Expression overlay: PAS-highFIB-highGCX vs. Naive-noFIB-highGCX. Pictured is the IPA signaling pathway, “Interleukin-10 signaling.” The following nodes are expanded to show member proteins: IL-10-downregulated extracellular proteins, -downregulated plasma membrane proteins, and -upregulated plasma membrane proteins. PAS-highFIB-highGCX versus naïve-noFIB-highGCX log_2_ fold-ratio values are overlayed upon the signaling pathway and are displayed as a simple color-intensity scale where magenta indicates increased and green indicates decreased protein abundance in PAS-highFIB-highGCX. More extreme differences in log_2_ fold-change between the comparison groups are reflected in the shading intensity. Colored proteins met the analysis cutoffs (>2-fold difference; Dunn’s pairwise comparisons, FDR<0.1); proteins shaded in grey are in the dataset but did not meet these cutoff requirements.
(TIF)

S6 Fig Communication between innate and adaptive immune cells ‐ Expression overlay: PAS-highFIB-highGCX vs. Naive-noFIB-highGCX. Pictured is the IPA signaling pathway, “Communication between Innate and Adaptive Immune Cells.” The IL1 node is expanded to show the member proteins that make up the node. PAS-highFIB-highGCX versus naïve-noFIB-highGCX log_2_ fold-ratio values are overlayed upon the signaling pathway and are displayed as a simple color-intensity scale where magenta indicates increased and green indicates decreased protein abundance in PAS-highFIB-highGCX. More extreme differences in log_2_ fold-change between the comparison groups are reflected in the shading intensity. Colored proteins met the analysis cutoffs (>2-fold difference; Dunn’s pairwise comparisons, FDR<0.1); proteins shaded in grey are in the dataset but did not meet these cutoff requirements.
(TIF)

S7 Fig Pathogen induced cytokine storm signaling pathway ‐ Expression overlay: PAS-highFIB-highGCX vs. Naive-noFIB-highGCX. Pictured is the IPA signaling pathway: “Pathogen Induced Cytokine Storm Signaling Pathway.” PAS-highFIB-highGCX versus naïve-noFIB-highGCX log_2_ fold-ratio values are overlayed upon the signaling pathway and are displayed as a simple color-intensity scale where magenta indicates increased and green indicates decreased protein abundance in PAS-highFIB-highGCX. More extreme differences in log_2_ fold-change between the comparison groups are reflected in the shading intensity. Colored proteins met the analysis cutoffs (>2-fold difference; Dunn’s pairwise comparisons, FDR<0.1); proteins shaded in grey are in the dataset but did not meet these cutoff requirements.
(TIF)

S8 Fig Role of macrophages, fibroblasts, and endothelial cells in rheumatoid arthritis (modified)–Expression overlay: PAS-highFIB-highGCX vs. Naive-noFIB-highGCX. Pictured is a modified “Role of Macrophages, Fibroblasts, and Endothelial Cells in Rheumatoid Arthritis” IPA signaling pathway. The IL-1 node is expanded to show the member proteins that make up the node. Additionally, the pathway has been cropped to exclude signaling in endothelial cells because the molecules and pathways are included in the rest of the pathway, and not shown are signaling pathways specific to rheumatoid arthritis. PAS-highFIB-highGCX versus naïve-noFIB-highGCX log_2_ fold-ratio values are overlayed upon the signaling pathway and are displayed as a simple color-intensity scale where magenta indicates increased and green indicates decreased protein abundance in PAS-highFIB-highGCX. More extreme differences in log_2_ fold-change between the comparison groups are reflected in the shading intensity. Colored proteins met the analysis cutoffs (>2-fold difference; Dunn’s pairwise comparisons, FDR<0.1); proteins shaded in grey are in the dataset but did not meet these cutoff requirements.
(TIF)

S9 Fig Wound healing signaling pathway. Pictured is the IPA signaling pathway “Wound Healing.” To see member proteins, the following nodes are expanded: “IL1”, “Pro-inflammatory cytokine”, “VEGF,” and “PDGF”.
(TIF)

S1 Table Clinical characteristics and included analyses for 63 patient samples. (CSV)

S2 Table FIB-GCX scoring. (CSV)

S3 Table Clinical characteristics for 54 patient cohort. (CSV)

S4 Table Categories of PAS, PAS-FIB-GCX, and pg/ml values of each immunomodulator for each patient. (CSV)

S5 Table Fig 2 PAS and naïve summary statistics. (CSV)

S6 Table Summary statistics of proteins in each PAS-FIB-GCX group. (CSV)

S7 Table Dunn’s pairwise analysis of Kruskal Wallis significant cytokines. (CSV)

S8 Table Ingenuity Pathway Analysis IDs for immunomodulators. (CSV)

## Abbreviations

GCX: Glycocalyx
PAS: previous abdominal surgeries
FIB: ECM fibers
MC: mesothelial cells
appy: appendectomy
SBO: small bowel obstruction
rA: patient reactive ascites exudate
