# Supplementary figures and images for: Mesothelial cell responses to acute appendicitis or small bowel obstruction reactive ascites: Insights into immunoregulation of abdominal adhesion
Source: PLoS One. 2025 Jan 8;20(1):e0317056. doi: 10.1371/journal.pone.0317056 (PMC11709316; doi:10.1371/journal.pone.0317056)

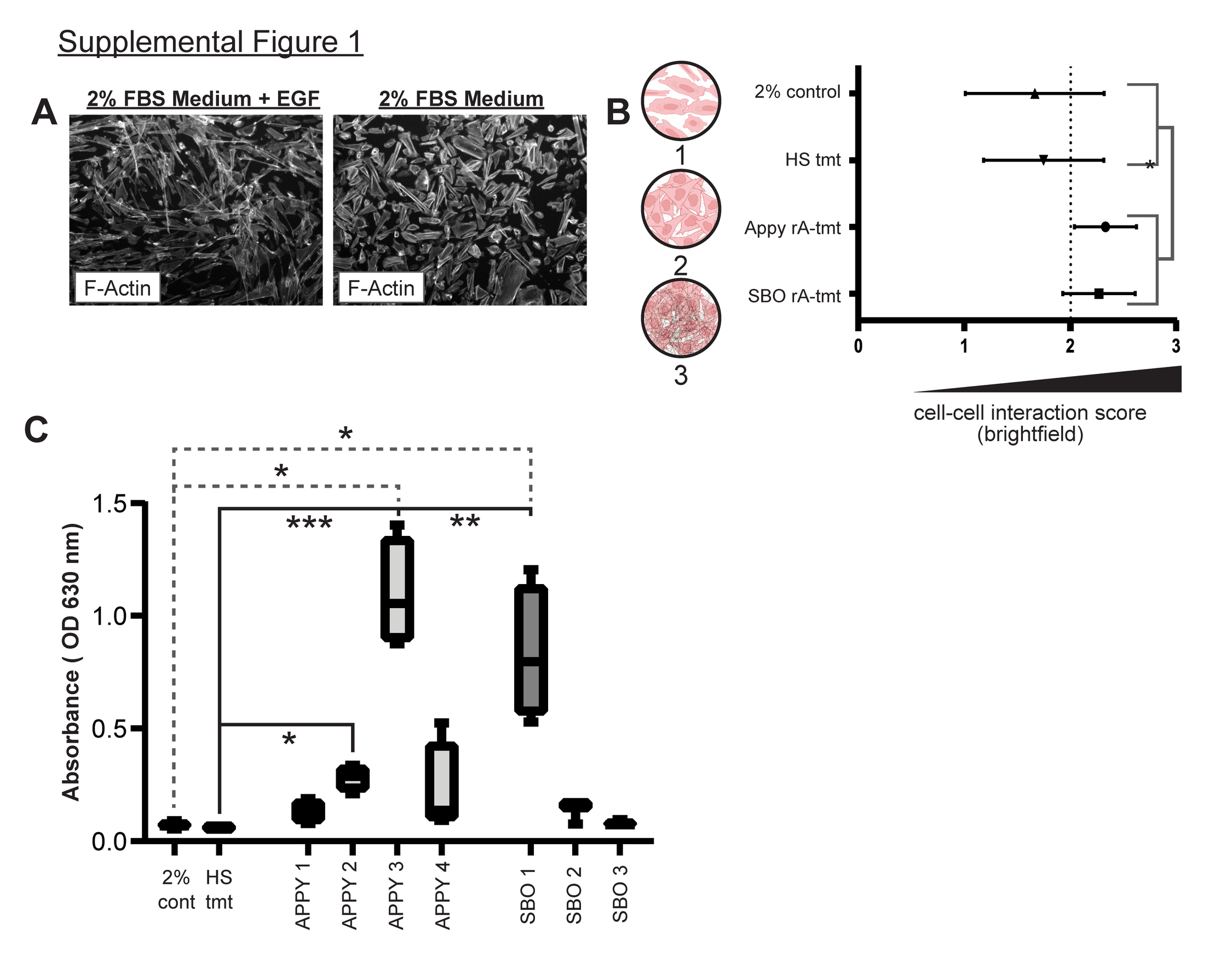

Supplement: S1 Fig — (A) Removing EGF from culture media drives mesothelial cells into a cuboidal-like morphology. Filamentous actin visualization with Phalloidin staining (Grayscale) on mesothelial cells in 2% media with EGF and 2% media without EGF. (B) Cartoon depicting cell-cell interaction scores corresponding to changes in MC morphology. Graph of cell-cell interaction scoring results following nested 1-way ANOVA of scoring from 3 independent experiments. Morphological changes were accessed by brightfield, and rA-treated MCs increased cell-to-cell interactions compared to controls (low interaction = 1, processes between cells = 2, dense thread-like fibers = 3, mean score ± SD: controls (2% Control and HS) 1.71 ± 0.46, appy and SBO rA-treated 2.31 ± 0.55, p < 0.05. (C) 48h-rA treated mesothelial cells were stained with Alcian blue at a pH of 2.5 to detect sulfated and carboxylated acid mucopolysaccharides and sulfated and carboxylated sialomucins. Relative Alcian blue staining was solubilized and quantified by microplate optical density (OD) at λ630nm. The two control conditions, 2% Control and HS showed very low levels of Alcian blue staining. Three of the rA showed significantly higher levels of Alcian blue staining after 48h culture compared to HS treatment controls: median optical density at 630nm ± SD: (HS, 0.0602 ± 0.005086; vs APPY 1, 0.1323 ± 0.04955 (ns); APPY 2, 0.2803 ± 0.05526 (* P<0.05); APPY 3, 1.097 ± 0.2398 (***P<0.001); APPY 4, 0.222 ± 0.2033 (ns); SBO 1, 0.8314 ± 0.2951 (**P<0.01); SBO 2, 0.1321 ± 0.04869 (ns); SBO 3, 0.07603 ± 0.01536 (ns). Results are graphed as a boxplot of median scores (interquartile range); *P<0.05; **P<0.01; ***P<0.001. (TIF) [file pone.0317056.s001.tif]

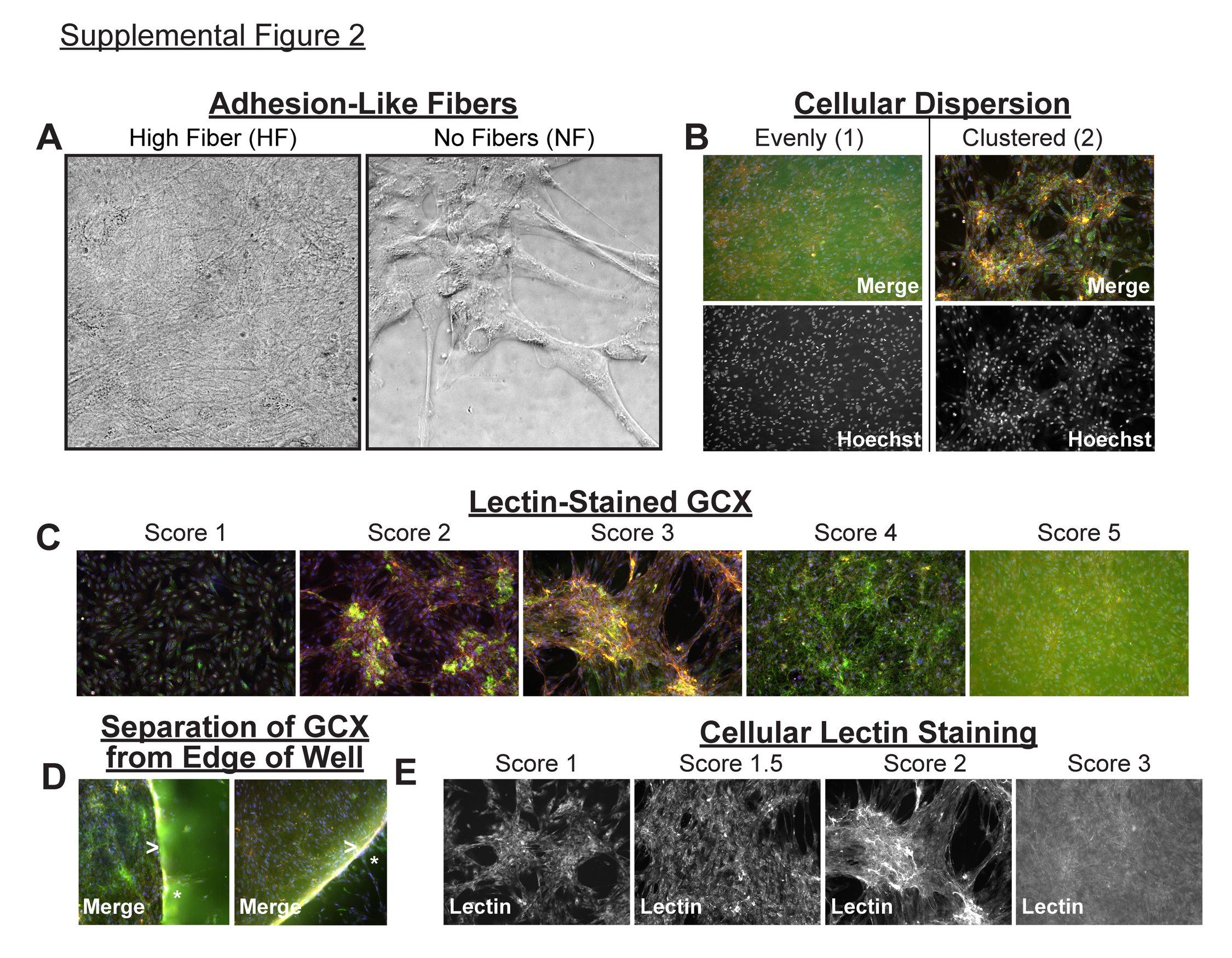

Supplement: S2 Fig — (A) Brightfield micrograph showing morphological changes in 48h rA-treated MCs from two separate patient fluids. Left panel illustrates the formation of ECM fibers that form over MCs occurring under treatment conditions of select patient rA fluids. The ECM fibers mask the overall morphology of the cells in contrast to the rA-treated MCs in the right-hand panel that did not form ECM fibers. (B-E) 48h following treatment with patient rA fluids, MCs were fixed and stained with the fluorescently labeled lectins: Concanavalin A– 488 and Wheat Germ Agglutinin– 594, and DAPI. (B) rA-treatment affected the overall dispersion of cells, and under select conditions, the cells were evenly distributed (left panels). In contrast, treatment with select rA-fluids caused the cells to form extensive clustered networks of cells (right hand panels). (C and D) As cells were not permeabilized, the fluorescent-lectin staining represents extracellular glycocalyx and extracellular matrix formation. (C) Lectin-stained fibers were scored at 5 levels corresponding to relative intensity of extracellular matrix produced by the cells. (D) In some instances, the GCX separated from the side of the culture well during fixation and staining. Caret represents the edge of the GCX, and the asterisks demark cells that stayed attached to the bottom of the tissue culture well. (E) Distinct patterns of extracellular lectin staining on the surface of the cells were observed and scored. A score of 1 showed staining that excluded the nuclear region in each cell, while a score of 2 showed extracellular staining that covered the cell evenly. Select rA treatments resulted in a mix of each staining pattern and were scored with 1.5. A score of 3 indicated that individually stained cells were difficult to ascertain due to the diffuse extracellular lectin staining of the glycocalyx. (TIF) [file pone.0317056.s002.tif]

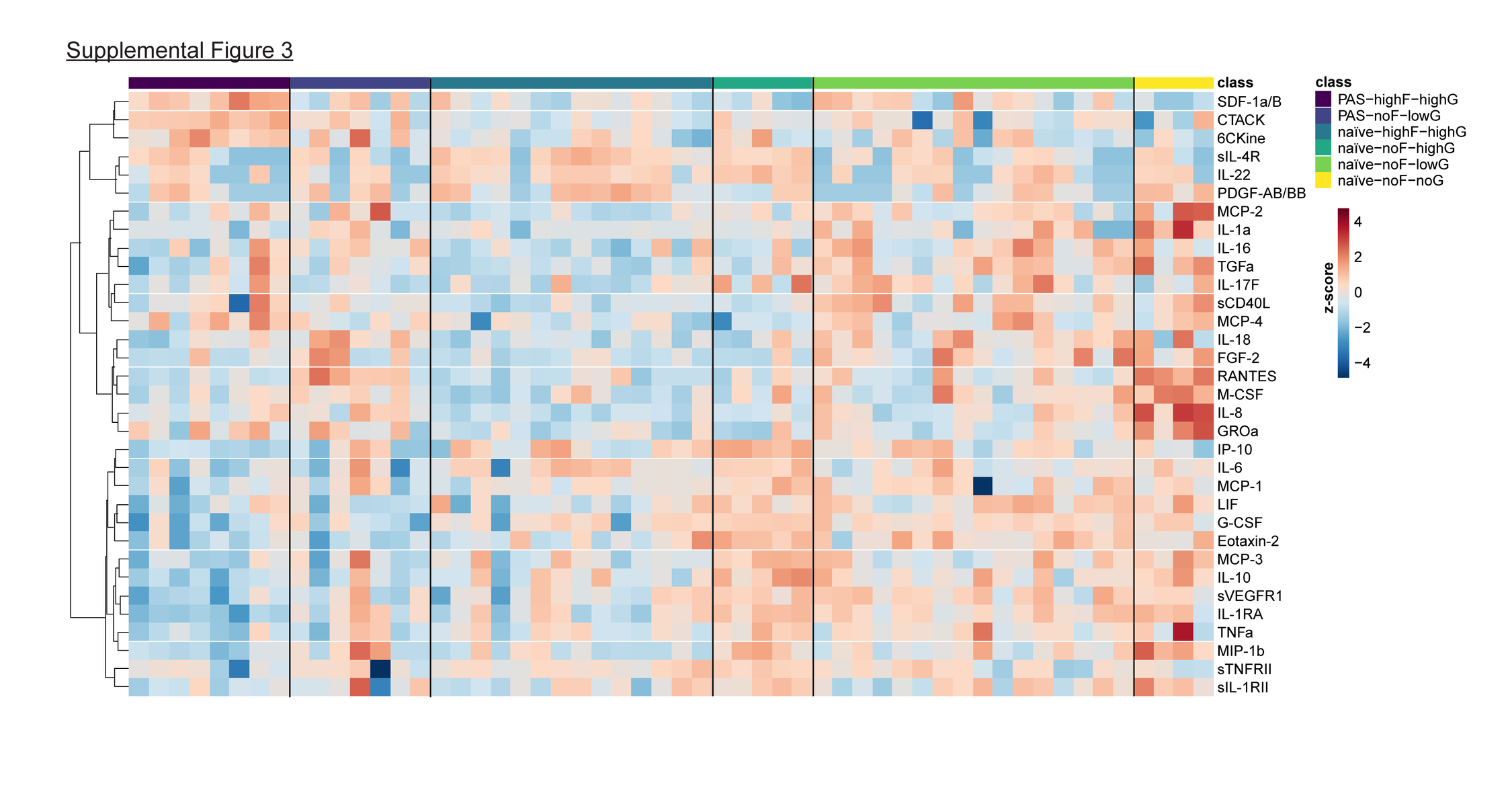

Supplement: S3 Fig — (A) Heatmap showing relative concentrations for 33 immunomodulators for each individual sample rA that significantly differed in PAS-Fiber-GCX samples (Kruskal Wallis; P<0.05). (TIF) [file pone.0317056.s003.tif]

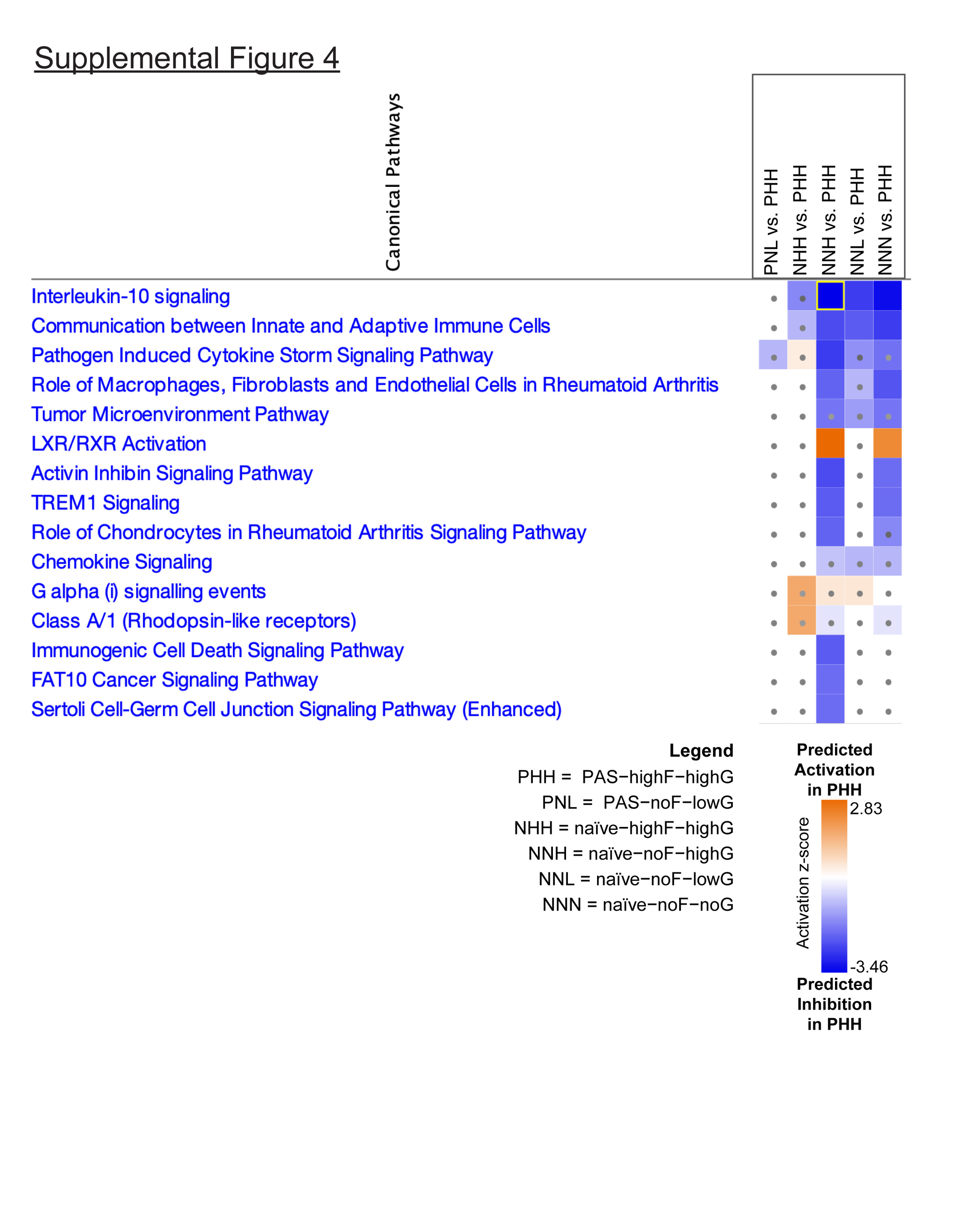

Supplement: S4 Fig — (A) Heatmap of activation z-scores for IPA canonical pathways showing predicted activation (z-score >2; orange) or inhibition (z-score <-2; purple) of pathways based on patterns of differential abundance observed for immunomodulators compared back to PAS-highFIB-highGCX. Boxes without color or grey dots demark pathway-dataset pairs where either no relationship or a non-significant z-score was calculated for that specific canonical pathway. Each column is a pairwise comparison of cytokine abundance in PAS-noFIB-lowGCX or naïve datasets as compared back to PAS-highFIB-highGCX. The top four pathways associated with these dataset pairs are “IL-10 signaling”, “Communication between Innate and Adaptive Immune Cells”, “Pathogen Induced Cytokine Storm Signaling Pathway”, and “Role of Macrophages, Fibroblasts, and Endothelial Cells in Rheumatoid Arthritis”. (TIF) [file pone.0317056.s004.tif]

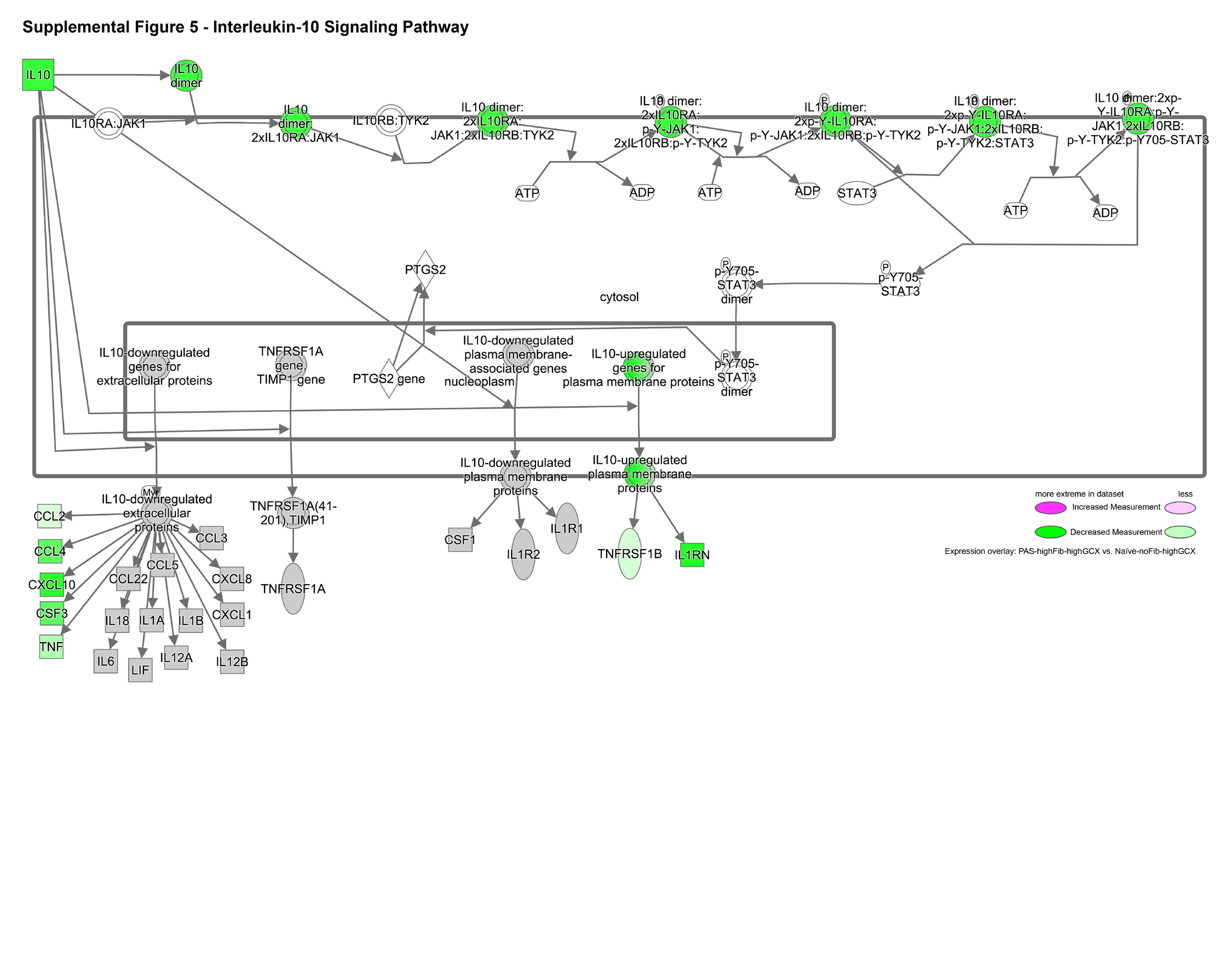

Supplement: S5 Fig — Pictured is the IPA signaling pathway, “Interleukin-10 signaling.” The following nodes are expanded to show member proteins: IL-10-downregulated extracellular proteins, -downregulated plasma membrane proteins, and -upregulated plasma membrane proteins. PAS-highFIB-highGCX versus naïve-noFIB-highGCX log2 fold-ratio values are overlayed upon the signaling pathway and are displayed as a simple color-intensity scale where magenta indicates increased and green indicates decreased protein abundance in PAS-highFIB-highGCX. More extreme differences in log2 fold-change between the comparison groups are reflected in the shading intensity. Colored proteins met the analysis cutoffs (>2-fold difference; Dunn’s pairwise comparisons, FDR<0.1); proteins shaded in grey are in the dataset but did not meet these cutoff requirements. (TIF) [file pone.0317056.s005.tif]

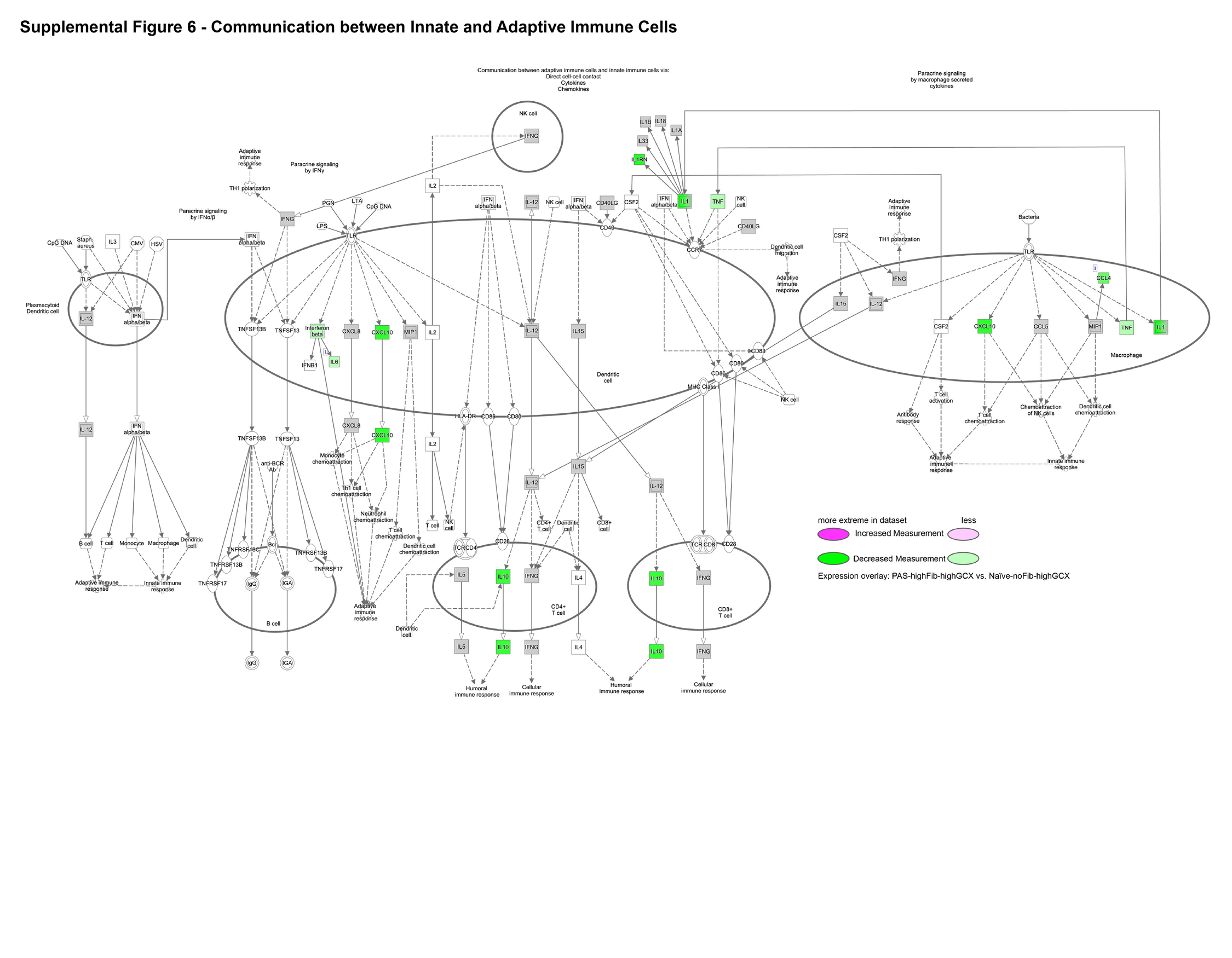

Supplement: S6 Fig — Pictured is the IPA signaling pathway, “Communication between Innate and Adaptive Immune Cells.” The IL1 node is expanded to show the member proteins that make up the node. PAS-highFIB-highGCX versus naïve-noFIB-highGCX log2 fold-ratio values are overlayed upon the signaling pathway and are displayed as a simple color-intensity scale where magenta indicates increased and green indicates decreased protein abundance in PAS-highFIB-highGCX. More extreme differences in log2 fold-change between the comparison groups are reflected in the shading intensity. Colored proteins met the analysis cutoffs (>2-fold difference; Dunn’s pairwise comparisons, FDR<0.1); proteins shaded in grey are in the dataset but did not meet these cutoff requirements. (TIF) [file pone.0317056.s006.tif]

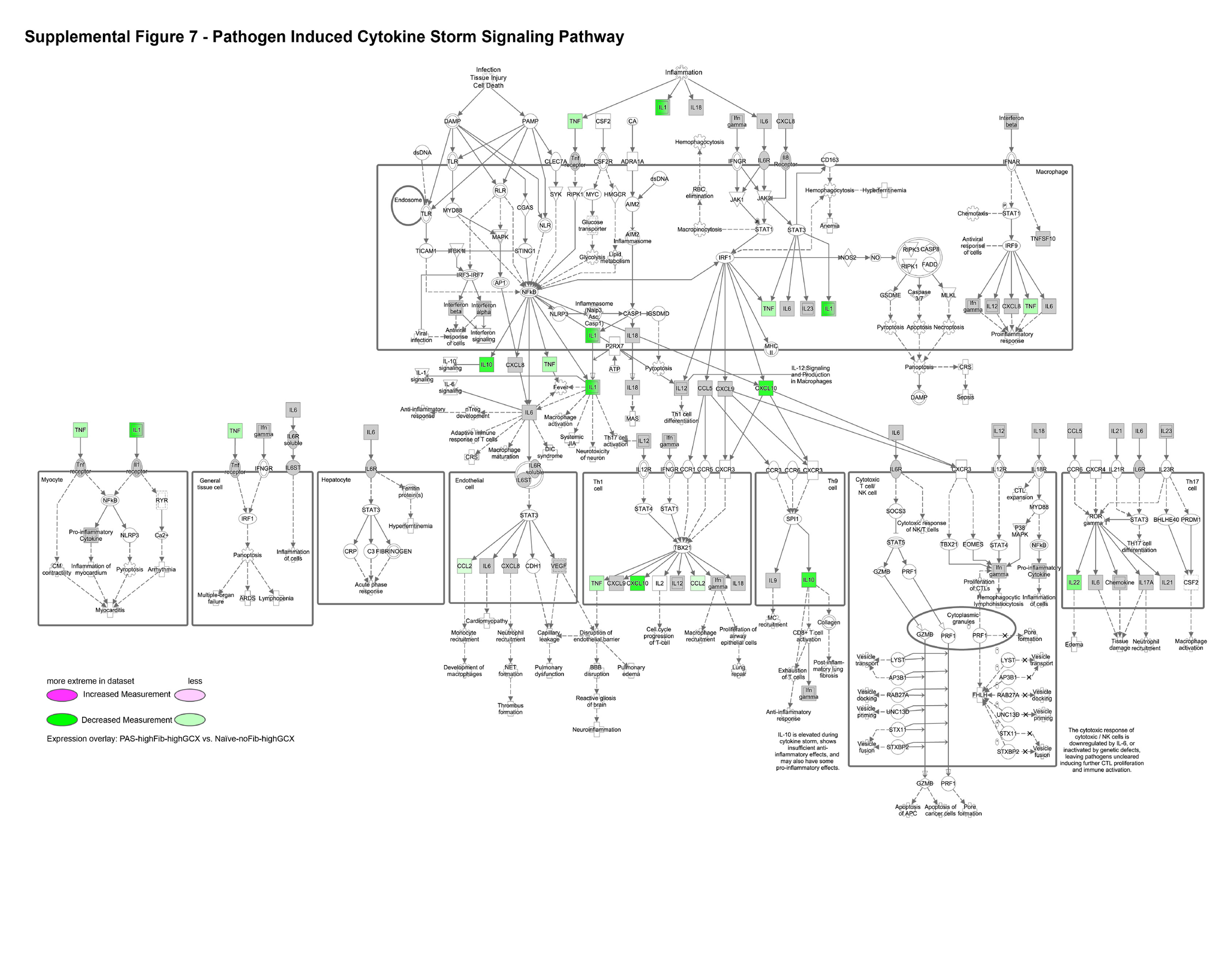

Supplement: S7 Fig — Pictured is the IPA signaling pathway: “Pathogen Induced Cytokine Storm Signaling Pathway.” PAS-highFIB-highGCX versus naïve-noFIB-highGCX log2 fold-ratio values are overlayed upon the signaling pathway and are displayed as a simple color-intensity scale where magenta indicates increased and green indicates decreased protein abundance in PAS-highFIB-highGCX. More extreme differences in log2 fold-change between the comparison groups are reflected in the shading intensity. Colored proteins met the analysis cutoffs (>2-fold difference; Dunn’s pairwise comparisons, FDR<0.1); proteins shaded in grey are in the dataset but did not meet these cutoff requirements. (TIF) [file pone.0317056.s007.tif]

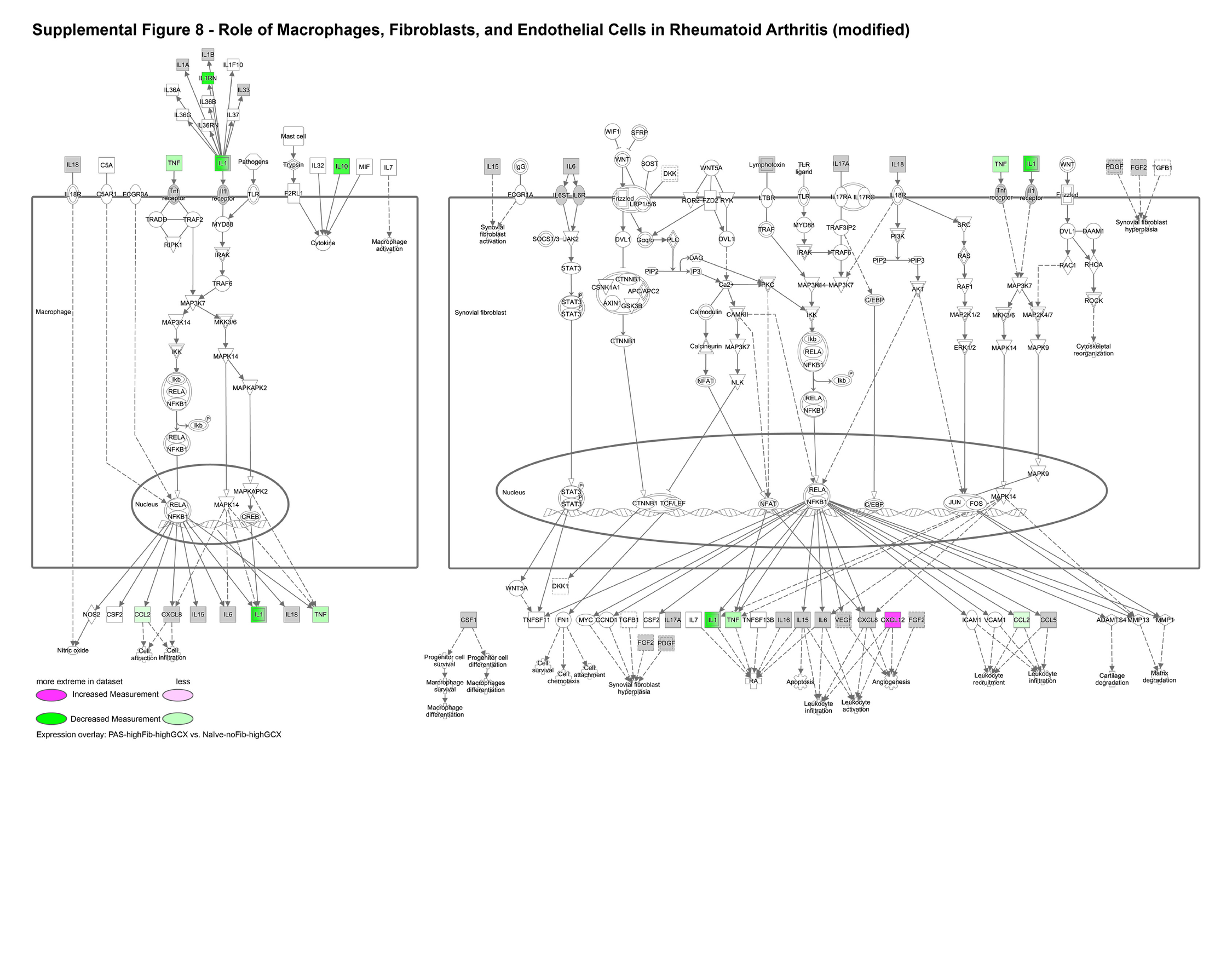

Supplement: S8 Fig — Pictured is a modified “Role of Macrophages, Fibroblasts, and Endothelial Cells in Rheumatoid Arthritis” IPA signaling pathway. The IL-1 node is expanded to show the member proteins that make up the node. Additionally, the pathway has been cropped to exclude signaling in endothelial cells because the molecules and pathways are included in the rest of the pathway, and not shown are signaling pathways specific to rheumatoid arthritis. PAS-highFIB-highGCX versus naïve-noFIB-highGCX log2 fold-ratio values are overlayed upon the signaling pathway and are displayed as a simple color-intensity scale where magenta indicates increased and green indicates decreased protein abundance in PAS-highFIB-highGCX. More extreme differences in log2 fold-change between the comparison groups are reflected in the shading intensity. Colored proteins met the analysis cutoffs (>2-fold difference; Dunn’s pairwise comparisons, FDR<0.1); proteins shaded in grey are in the dataset but did not meet these cutoff requirements. (TIF) [file pone.0317056.s008.tif]

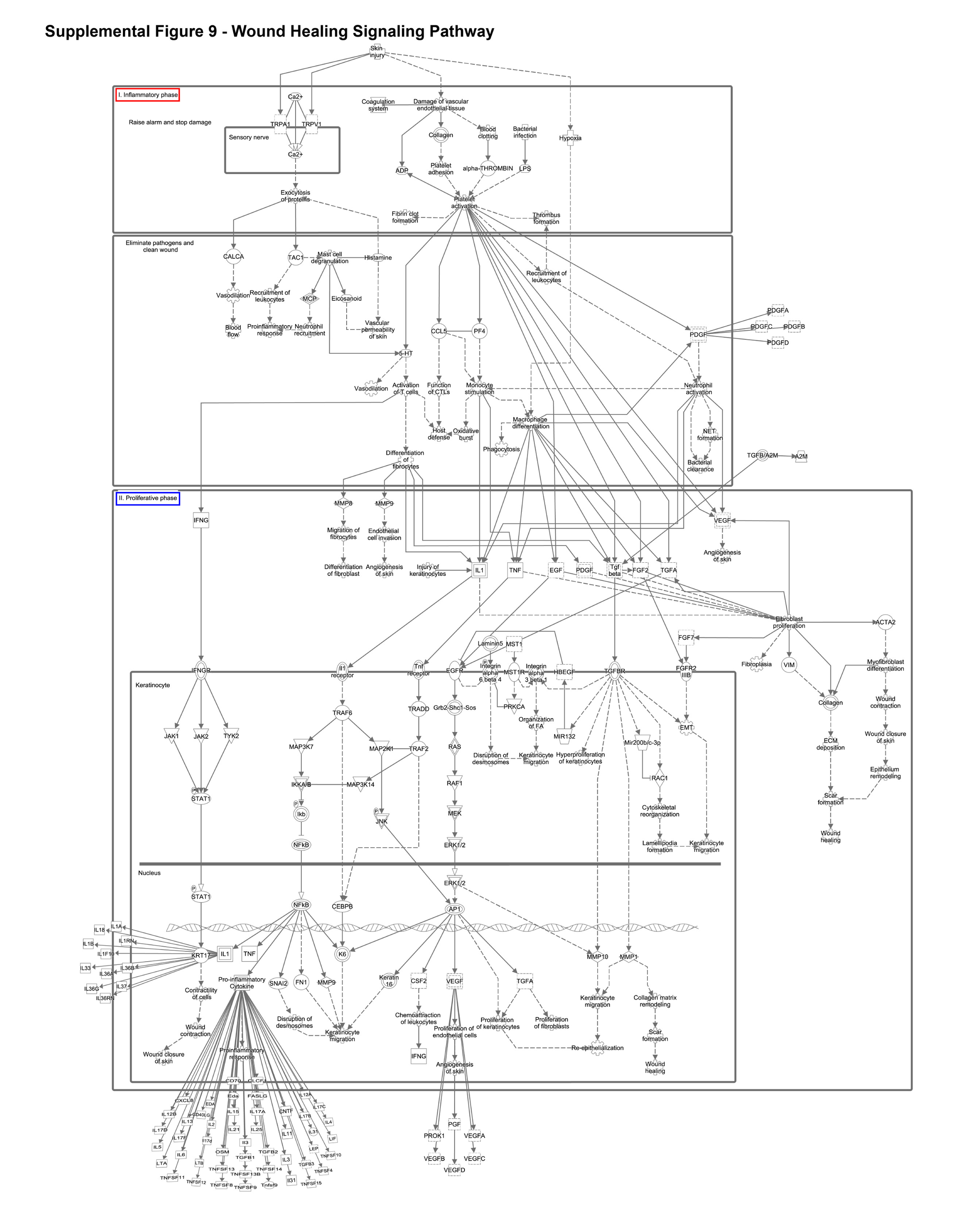

Supplement: S9 Fig — Pictured is the IPA signaling pathway “Wound Healing.” To see member proteins, the following nodes are expanded: “IL1”, “Pro-inflammatory cytokine”, “VEGF,” and “PDGF”. (TIF) [file pone.0317056.s009.tif]
